# Parental occupations at birth and risk of adult testicular germ cell tumors in offspring: a French nationwide case–control study

**DOI:** 10.3389/fpubh.2023.1303998

**Published:** 2024-01-16

**Authors:** Adèle Paul, Aurélie M. N. Danjou, Floriane Deygas, Margot Guth, Astrid Coste, Marie Lefevre, Brigitte Dananché, Hans Kromhout, Johan Spinosi, Rémi Béranger, Olivia Pérol, Helen Boyle, Christel Hersant, Vanessa Loup-Cabaniols, Ségolène Veau, Louis Bujan, Ann Olsson, Joachim Schüz, Béatrice Fervers, Barbara Charbotel

**Affiliations:** ^1^UMRESTTE (Epidemiological Research and Surveillance Unit in Transport, Occupation and Environment), Lyon 1 University, Eiffel University, Lyon, France; ^2^Department of Occupational Health, AMEBAT, Nantes, France; ^3^Environment and Lifestyle Epidemiology Branch, International Agency for Research on Cancer/World Health Organization, Lyon, France; ^4^Département Prévention, Cancer et Environnement, Centre Léon Bérard, Lyon, France; ^5^Department of Medical Oncology, Centre Léon Bérard, Lyon, France; ^6^Department of Environmental Epidemiology, Institute or Risk Assessment Sciences, Utrecht University, Utrecht, Netherlands; ^7^Direction Santé Travail, Santé Public France, Saint Maurice, France; ^8^Univ Rennes, CHU Rennes, Inserm, EHESP, Irset (Institut de Recherche en Santé, Environnement et Travail), Rennes, France; ^9^Maternité, CHRU Nancy, Nancy, France; ^10^Department of Reproductive Biology, CECOS, University Hospital of Montpellier, Montpellier, France; ^11^Department of Reproductive Medicine and Biology, CECOS, CHU Rennes, Rennes, France; ^12^DEFE (Développement Embryonnaire, Fertilité, Environnement) INSERM 1202 Universités Montpellier et Toulouse 3, CECOS Hôpital Paule de Viguier, CHU de Toulouse, Toulouse, France; ^13^Fédération Française des CECOS, Paris, France; ^14^Inserm UA1296 Radiations: Défense, Santé, Environnement, Lyon, France; ^15^CRPPE Lyon (Centre Régional de Pathologies Professionnelles et Environnementales), Hospices Civils de Lyon, Lyon, France

**Keywords:** testicular cancer, testicular germ cell tumor, parental occupational exposure, prenatal exposure, parental job, parental occupation

## Abstract

**Background:**

Testicular germ cell tumors (TGCT) are the most frequent cancer in young men in developed countries. Parental occupational exposures during early-life periods are suspected to increase TGCT risk. The objective was to estimate the association between parental occupations at birth and adult TGCT.

**Methods:**

A case–control study was conducted, including 454 TGCT cases aged 18–45 from 20 French university hospitals, matched to 670 controls based on region and year of birth. Data collected from participants included parental jobs at birth coded according to the International Standard Classification of Occupation—1968 and the French nomenclature of activities—1999. Odds ratios (OR) for TGCT and 95% confidence intervals (CI) were estimated using conditional logistic regression, adjusting for TGCT risk factors.

**Results:**

Paternal jobs at birth as service workers (OR = 1.98, CI 1.18–3.30), protective service workers (OR = 2.40, CI 1.20–4.81), transport equipment operators (OR = 1.96, CI 1.14–3.37), specialized farmers (OR = 2.66, CI 1.03–6.90), and maternal jobs as secondary education teachers (OR = 2.27, CI 1.09–4.76) or in secondary education (OR = 2.35, CI 1.13–4.88) were significantly associated with adult TGCT. The risk of seminoma was increased for the above-mentioned paternal jobs and that of non-seminomas for public administration and defence; compulsory social security (OR = 1.99, CI 1.09–3.65); general, economic, and social administration (OR = 3.21, CI 1.23–8.39) for fathers; and secondary education teacher (OR = 4.67, CI 1.87–11.67) and secondary education (OR = 3.50, CI 1.36–9.01) for mothers.

**Conclusion:**

Some paternal jobs, such as service workers, transport equipment operators, or specialized farmers, and maternal jobs in secondary education seem to be associated with an increased risk of TGCT with specific features depending on the histological type. These data allow hypotheses to be put forward for further studies as to the involvement of occupational exposures in the risk of developing TGCT, such as exposure to pesticides, solvents, or heavy metals.

## 1 Introduction

Testicular germ cell tumors (TGCT) are the most frequent cancer diagnosed in young men (aged 15–45) and represent 94–98% of testicular cancers (1–3). Their incidence rate has been increasing over the past 40 years, in particular in developed countries and among Caucasian populations (2, 4, 5). To date, only genetic polymorphisms and familial and personal history of testicular cancer are considered established risk factors (6, 7). Yet, the spatial variations of TGCT (8), the incidence increase of TGCT in many countries, and its rise in migrant populations to more developed industrialized countries (9) are consistent with a possible role of the environment in the development of TGCT.

The hypothesis of an early origin of the disease has been suggested, reinforced by the young age at diagnosis and the fact that the main TGCT histological subtypes, seminomas and non-seminomas, derive from a common precursor present in children (germ cell neoplasia *in situ*, (GCNIS)) originating from *in utero* transformation of primordial germ cells blocked in their maturation, as well as by a supposed link with specific congenital malformations (cryptorchidism and hypospadias) and altered spermatogenesis, grouped under the “Testicular Dysgenesis Syndrome” (3, 6, 10–12). Intra-uterine period, especially in the first trimester of pregnancy, is critical for gonadal development (13–16). It is a sensitive window of exposure for the foetus to parental exposures (17, 18), in particular to endocrine-disrupting compounds (EDC) that affect the androgen/oestrogen balance (13–15, 19–21). While most studies on parental exposures and the risk of TGCT investigate isolated endocrine disruptors with long half-lives (22), in particular chronic exposure (22), at low doses (22, 23), there are also arguments for the impact of exposures to endocrine disruptors with short half-lives (22, 24) and for the combined effects of multiple EDC with possible additive, antagonist, or synergistic effects (22). Such exposures could induce downregulation of androgen and oestrogen receptor signaling (25, 26), oxidative damage and apoptosis in testes observed in animal and *in vitro* studies (27), inhibition of DNA synthesis in testes seen in animal studies (28, 29) or act as a tumor promoter through the inhibition of intercellular communication (30), which may contribute to GCNIS development in the early stages of pregnancy (13, 14, 16, 31, 32). The hypothesis of prenatal and/or early-life exposures is now widely accepted (33–35), including parental exposures before birth as risk factor candidates for TGCT in the offspring.

In France, although exposure to carcinogenic, mutagenic, and reprotoxic (CMR) substances at work has been decreasing (36), close to 14% of employees were occupationally exposed to one or more CMR agents in 2003, and still over 10% in 2010, with more than 3% exposed to multiple CMR agents at work (37). In Europe, certain job categories and sectors of activity appear to be more exposed to CMRs, such as workers in the production or application of pigments, resins, cutting fluids, adhesives, pesticides, cleaning products, rubber, plastics, textiles, pharmaceuticals, cosmetics, and in agriculture, metallurgy, or food processing industry (38). Moreover, a Dutch job exposure matrix estimated that between 1996 and 2006, 29% of jobs were possibly (17%) or probably (12%) exposed to one or several endocrine-disrupting chemicals (39). Cohort or case–control studies have suggested a link between certain parental jobs at birth and the risk of developing TGCT (40–42). However, these studies are very few in number and showed contradictory and inconsistent results that may differ according to the histological subtype (42–46).

This study aimed to estimate the association between parental employment at birth and the risk of developing adult TGCT, according to the seminoma or non-seminoma histological subtypes, in the offspring.

## 2 Methods

### 2.1 Study design

The study analyzed data from a multicentre prospective case–control study conducted between January 2015 and April 2018 in 20 university hospital centres in Metropolitan France. The detailed study protocol has been previously published (47, 48). The study involved patients diagnosed with primary TGCT, aged 18–45, who were referred for semen preservation to the regional sperm banks in the Centers for the Study and Storage of Human Sperm and Eggs (CECOS). Controls were frequency matched to cases by year of birth (±3 years) and hospital centre administrative region. Not being born in metropolitan France and having a personal history of testicular cancer or cryptorchidism in controls were exclusion criteria. Two groups of controls were identified: group A controls were sperm donors recruited in CECOS and partners, with normal sperm production, of women consulting for infertility recruited in assisted reproduction treatment centres; group B controls were partners of women in high-risk pregnancy services at the hospital centre. Written consent was required. A two-stage recruitment process of case/control’s mothers/relatives was performed, asking participants who had consented for permission to contact their biological mother or, if not available, the closest relative alive. Upon the agreement of the participant, the mother/relative was invited to participate in the study.

Participants’ interviews were conducted through a 90-min phone call by trained investigators (IPSOS Company) blinded to the case–control status with a structured, pretested, and computer-assisted questionnaire (47, 49). The questionnaire was tested in the pilot study among 45 subjects (28 cases and 17 controls) and 23 mothers (49). Interviewers were provided with a field guide and were trained in the completion of the questionnaire in order to ensure consistency in data collection. Information on parental occupation at birth, socio-economic status, birth characteristics, medical history, and lifestyle factors were collected. Upon consent from participants and their mothers, interviews were conducted with mothers under the same conditions. Information collected from mothers included their work history and that of the son’s biological father. After signing the informed consent, cases and controls, as well as the subjects’ mothers, were given a document to prepare for the telephone interview, especially regarding their professional and residential history. Moreover, they were invited to bring their health record to the interview, as some questions covered items recorded in the health record (e.g., birth weight, birth height).

The selection of the study population is described in detail in Supplementary Figure S1. Briefly, of the 550 TGCT cases recruited in the study, 30 were excluded according to the inclusion and exclusion criteria based on the review of the pathology reports. From the 447 group A and 370 group B controls recruited, one group B control not born in Metropolitan France and 13 controls (eight group A and five group B) who reported personal history of cryptorchidism were excluded. Overall, 168 subjects did not complete the interview (48 TGCT cases, 46 group A controls, and 74 group B controls) for the following reasons: 44 refused to participate; 123 were unreachable after three attempts; and 1 person was deceased prior to the interview. Finally, 31 participants (18 TGCT cases, 13 controls) were excluded because only their mothers/relatives had completed the interview.

The study population finally included 1,124 eligible participants: 454 TGCT cases (219 seminomas, 191 non-seminomas, 1 unknown histological subtype, and 43 subjects with missing pathology reports), 384 group A controls, and 286 group B controls (Table 1).

**Table 1:** Characteristics of TGCT cases and controls (group A and group B), case–control study, *N* = 1,124, France, 2015–2018.

	TGCT cases (*n* = 454)	Group A controls (*n* = 384)	Group B controls (*n* = 286)
	*n* (%)	*n* (%)	*n* (%)
Age at diagnosis (cases)/inclusion (controls) (years)			
≤25	64 (14.1)	20 (5.2)	12 (4.2)
26–30	106 (23.4)	80 (20.8)	64 (22.4)
31–35	113 (24.9)	131 (34.1)	108 (37.8)
36–40	85 (18.7)	95 (24.7)	67 (23.4)
≥41	43 (9.5)	58 (15.1)	35 (12.2)
Missing	43 (9.5)	0 (0.0)	0 (0.0)
Year of birth			
<1975	37 (8.2)	33 (8.6)	17 (5.9)
1975–1979	80 (17.6)	82 (21.4)	46 (16.1)
1980–1984	117 (25.8)	119 (31.0)	99 (34.6)
1985–1989	130 (28.6)	105 (27.3)	98 (34.3)
1990–1994	70 (15.4)	40 (10.4)	23 (8.0)
1995–1999	20 (4.4)	5 (1.3)	3 (1.1)
Education			
Secondary	180 (39.7)	137 (35.7)	66 (23.1)
1- to 2-year university degree	97 (21.4)	91 (23.7)	57 (19.9)
>3-year university degree	128 (28.2)	113 (29.4)	121 (42.3)
Other	48 (10.6)	43 (11.2)	42 (14.7)
Missing	1 (0.2)	0 (0.0)	0 (0.0)
Smoking status			
Former smoker	102 (22.5)	99 (25.8)	72 (25.2)
Current smoker	147 (32.4)	116 (30.2)	69 (24.1)
Never smoker	205 (45.2)	169 (44)	145 (50.7)
Geographic origin			
French by birth	449 (98.9)	380 (99.0)	281 (98.3)
French by acquisition	5 (1.1)	4 (1.0)	5 (1.8)
Birth weight (g)			
<2,500	25 (5.5)	20 (5.2)	13 (4.6)
2,500–4,000	356 (78.4)	318 (82.8)	243 (85)
≥4,000	46 (10.1)	32 (8.3)	26 (9.1)
Missing	27 (6.0)	14 (3.7)	4 (1.4)
Gestational age (weeks)			
≤36	32 (7.1)	23 (6.0)	11 (3.9)
>36	415 (91.4)	358 (93.2)	271 (94.8)
Missing	7 (1.5)	3 (0.8)	4 (1.4)
Admitted to neonatology service at birth			
No	365 (80.4)	339 (88.3)	256 (89.5)
Yes	48 (10.6)	29 (7.6)	25 (8.7)
Missing	41 (9.0)	16 (4.2)	5 (1.8)
Born from multiple pregnancy			
No	433 (95.4)	375 (97.7)	277 (96.9)
Yes	21 (4.6)	8 (2.1)	9 (3.2)
Missing	0 (0.0)	1 (0.3)	0 (0.0)
Birth order			
First	213 (46.9)	168 (43.8)	137 (47.9)
Second	163 (35.9)	135 (35.2)	89 (31.1)
Third	63 (13.9)	51 (13.3)	42 (14.7)
Fourth and more	15 (3.3)	30 (7.8)	18 (6.3)
Sibship size			
1	25 (5.5)	36 (9.4)	23 (8.0)
2	192 (42.3)	139 (36.2)	115 (40.2)
3	153 (33.7)	116 (30.2)	90 (31.5)
≥4	84 (18.5)	92 (24)	58 (20.3)
Missing	0 (0.0)	1 (0.3)	0 (0.0)
Personal history of inguinal hernia			
No	414 (91.2)	362 (94.3)	275 (96.2)
Yes	39 (8.6)	22 (5.7)	10 (3.5)
Missing	1 (0.2)	0 (0.0)	1 (0.4)
Personal history of hypospadias			
No	450 (99.1)	382 (99.5)	284 (99.3)
Yes	3 (0.7)	2 (0.5)	2 (0.7)
Missing	1 (0.2)	0 (0.0)	0 (0.0)
Personal history of testicular trauma			
No	387 (85.2)	351 (91.4)	260 (90.9)
Yes	67 (14.8)	33 (8.6)	26 (9.1)
Family history of TGCT			
No	419 (92.3)	375 (97.7)	276 (96.5)
Yes	33 (7.3)	7 (1.8)	9 (3.1)
Missing	2 (0.4)	2 (0.5)	1 (0.4)
Family history of cryptorchidism			
No	422 (92.9)	369 (96.1)	276 (96.5)
Yes	28 (6.2)	10 (2.6)	8 (2.8)
Missing	4 (0.9)	5 (1.3)	2 (0.7)
Age at voice change (years)			
<12	16 (3.5)	17 (4.4)	9 (3.2)
12–16	375 (82.6)	301 (78.4)	231 (80.8)
>16	53 (11.7)	56 (14.6)	38 (13.3)
Missing	10 (2.2)	10 (2.6)	8 (2.8)
Cannabis use in young adulthood (18–25 years)			
No	262 (57.7)	219 (57.0)	174 (60.8)
Yes	192 (42.3)	164 (42.7)	112 (39.2)
Missing	0 (0.0)	1 (0.3)	0 (0.0)
Frequency of cannabis use in young adulthood (18–25 years)			
Never	262 (57.7)	219 (57.0)	174 (60.8)
Less than once/month	62 (13.7)	62 (16.1)	45 (15.7)
≥1 time/month	23 (5.1)	25 (6.5)	21 (7.3)
Once/week	41 (9.0)	33 (8.6)	21 (7.3)
Once/day	66 (14.5)	44 (11.5)	25 (8.7)
Missing	0 (0.0)	1 (0.3)	0 (0.0)

The study received ethical approval from the French Ethics Committee (ref. no. A14-94), the French national agency for medicines and health products safety (ref. no. 140184B-12), and the IARC Ethics Committee (ref. no. 14–26), and was declared to the Commission nationale de l’Informatique et des Libertés (MR-001, ref. no. 2016–177), as well as registered at http://www.clinicaltrials.gov (NCT02109926).

### 2.2 Ascertainment of TGCT cases

TGCT cases were histologically confirmed from pathology reports (92.2%) and serum tumor markers (84%) by a TGCT expert (HB) (*N* = 550). Only patients with GCNIS-related tumor classified as seminomatous or non-seminomatous according to the International Classification of Disease for Oncology and the World Health Organization (WHO) classification of tumors of the urinary system and male genital organs were included (3, 50). TGCT cases with missing pathology report (7.8%, *N* = 43) were included as cases since the proportion of false-positive TGCT was low (5.1%).

### 2.3 Job and industry coding

Parental jobs and industries at birth, reported by the participants and by the mothers, were encoded by an industrial hygienist, blinded to the case–control status of participants, using the International Standard Classification of Occupations 1968 (ISCO-68) for jobs (51) and the French nomenclature of activity 1999 (NAF-1999) for industries (52). The term “job” refers to the profession carried out by the subject (e.g., cook, nurse, production worker, etc.). The term “industry” is used to characterize the sector of activity in which the subject works (e.g., in a restaurant, a public administration, a hospital, a laboratory, in the plastics industry, or the food industry). In this article, the combination of the two can be referred to as “occupation.” These two nomenclatures were chosen among the official nomenclatures classically used in epidemiology in accordance with the specificity of the study population, combining information on jobs and industries. Parents’ occupations were coded on the basis of the job title and main task described on the one hand by the participants and on the other by their mother or a family member. If in doubt, the most relevant code was assigned after discussion among experts in occupational exposure research (BC, BD, and JSp). Coding of mother-reported parental employment and participant-reported parental employment was done independently and blinded to each other’s reported data. We focused on the parental employment at birth as a proxy for the periconceptional and perinatal periods. Agreement between parental employment at birth reported by the participants whose mothers had agreed to participate and the ones reported by their mothers was estimated using Cohen’s kappa coefficient (53).

As military personnel are not classified in ISCO-68, the code was assigned on the basis of their main task (e.g., military doctor classified as 0–61.05 “general physician”). Similarly, apprenticeship students were coded for the job they trained for (e.g., baker’s apprentice, classified as 7–76.10, “Baker, general”).

### 2.4 Statistical analysis

Odds ratios (OR) and 95% confidence intervals (CI) for adult TGCT were estimated for job and industry titles of maternal and paternal jobs at birth (reference category: not being employed in this job category), using conditional logistic regression models for frequency-matched case–control sets (54). ISCO-68 and NAF-1999 codes with various digit levels were analyzed and we retained in the analysis job and industry titles for which at least five cases’ mothers/fathers and five controls’ mothers/fathers had been employed. This resulted in the following number of analyzed jobs in cases/controls in crude analysis: for fathers, based on ISCO-68, 416/647 for one and two digits, 408/620 for three digits, and 365/561 for four and five digits, and based on NAF-1999, 376/592 for two digits, 342/530 for three digits, and 288/455 for four digits; for mothers, based on ISCO-68, 428/652 for one and two digits, 426/648 for three digits, and 402/626 for four and five digits, and based on NAF-1999, 425/640 for two digits, 394/597 for three digits, and 357/533 for four digits. All models were conditioned for matching factors (hospital’s administrative region and birth year). As some cases could not be associated with controls and vice versa, in individuals, we performed frequency matching on year of birth, grouped into 5-year categories, and region of recruitment (55).

Perinatal covariates considered for adjustment were: birth weight (<2,500, 2,500–4,000, >4,000 g); gestational age (≤36 weeks, >36 weeks); birth order (first, second, third, fourth, and more); sibship size (one, two, three, four, and more brothers and sisters); and being born from a multiple pregnancy (yes, no). We also identified family history of testicular cancer (yes, no), family history of cryptorchidism (yes, no), and several individual factors: personal history of testicular trauma (yes, no); tobacco smoking (yes, no); cannabis use (yes, no); cannabis frequency use (less than once a month, at least once a month, once a week, once a day, never) during young adulthood (18–25 years old); and age at voice change (<12, 12–16, >16 years old) as a proxy for delayed puberty. Separate models were run for each covariate. If the value of p of Wald statistic was <0.20, in the next step, the respective covariate was included in a joint model. This joint model was used iteratively, and one by one, the covariate with the highest value of p was removed from the model. The final model included only the covariates statistically significantly associated with the outcome (value of ps <0.05): sibship size, birth from multiple pregnancies, personal history of testicular trauma, family history of testicular cancer, and family history of cryptorchidism.

OR and CI for adult TGCT were also stratified based on the histological subtypes, such as seminomas and non-seminoma. Heterogeneity of associations was tested using the likelihood ratio test of polytomous logistic regression for matched case–control studies (56). Cases with missing histological subtype (*N* = 44), missing report (*N* = 43), and unknown subtype (*N* = 1) were excluded from this analysis.

As sensitivity analyses, the associations between parental job and industry titles at birth and adult TGCT risk were investigated, excluding cases with personal history of cryptorchidism (*N* = 40) and excluding TGCT cases not confirmed by pathology reports (*N* = 43). Finally, OR and CI were estimated for parental jobs at birth reported by the mother or a close relative.

Due to the broad 5-year age strata for matching, additional analyses with adjustment for age (as a continuous variable) were performed for overall TGCT. The age at diagnosis (cases) and age at inclusion (controls) were introduced continuously into the models, and matching was based solely on region.

OR were considered statistically significant when the 95% confidence interval did not include the value of 1.00. Data analysis was performed using SAS statistical software version 9.4 (SAS Institute Inc., Cary, NC, USA).

## 3 Results

### 3.1 Characteristics of the study population

Eighteen (1.6%) participants were excluded from the risk analysis due to missing information on adjustment factors. The two-stage recruitment process of case/control mothers/relatives in the present study resulted in a 50% participation rate among mothers/relatives.

The age of cases at diagnosis was lower than that of controls at inclusion (mean age [±SD]: 31.9 years [±6.1] in cases versus 33.6 years [±5.4]; value of *p* <0.001). Most of the participants were first born (43.8–47.9%), born at term (90.8–94.1%), and had a normal weight at birth (2500–4,000 g, 78.4–85.0%). There were more cases than controls born from a multiple pregnancy (4.6% (*N* = 21) in cases versus 2.1% (*N* = 8) and 3.2% (*N* = 9) in controls). Family history of testicular cancer was more frequent among TGCT cases than controls (7.3% (*N* = 33) versus 1.8% (*N* = 7) and 3.2% (*N* = 9)), as well as testicular trauma [14.8% (*N* = 67) versus 8.6% (*N* = 33) and 9.1% (*N* = 26)] and family history of cryptorchidism [6.2% (*N* = 28) versus 2.6% (*N* = 10) and 2.8% (*N* = 8)]. Few participants had a personal history of hypospadias [0.7% (*N* = 3) versus 0.5% (*N* = 2) and 0.7% (*N* = 3)]. There was no difference between TGCT cases and controls in education level, geographic origin, smoking status, or age at voice change.

Data on fathers’ and mothers’ occupations at birth were obtained from 570 mothers and 8 relatives (*N* = 578, 50% participation rate) and used to assess the validity of information obtained directly from the 578 corresponding participants involved: 304 TGCT cases, 145 group A controls and 129 group B controls (Supplementary Table S1). Cohen’s kappa values ranged from moderate agreement for the most precise coding levels (four-digit NAF and five-digit ISCO codes), with a minimum of 0.50 for paternal four- and five-digit ISCO; to almost perfect agreement for less precise coding levels (two-digit NAF and one-digit ISCO codes), with a maximum of 0.85 for maternal two-digit NAF). It was therefore decided to carry out the main analysis with the data reported by the participants, since they were more exhaustive.

Of the 1,124 participants, 1,042 reported employment at birth for their fathers (95.4%) and 751 for their mothers (66.8%). Based on the information collected, a two-digit ISCO-68 code was assigned to 1,042 paternal jobs (92.7%) and 734 maternal jobs (65.3%) and a two-digit NAF code to 957 paternal jobs (85.1%) and 719 maternal jobs (64%). Overall, the assessment resulted in ISCO codes up to five digits and NAF codes up to four digits for 909 (87.2%) and 725 (83.6%) paternal jobs, and 682 (92.9%) and 546 (75.9%) maternal jobs. Therefore, as the total number of analyzed subjects decreases with the number of ISCO-68 and NAF digits used for defining broader and more specific jobs, the underlying numbers of the results for the more specific jobs within a broader job category do not add up to the total of the numbers of the results of the broader job category. Comparing the categories of parental employment at birth according to whether or not the mother had participated in the study, we observe that the interviewed mothers were themselves, as well as the biological father, in more favorable employment conditions (Supplementary Table S2).

### 3.2 TGCT risk and father’s employment

“Service workers (5),” the broad category gathering cooks, bartenders, waiters, and protective service workers (such as firefighters, policemen, etc.), for example, were the only main job group for which a statistically significant association between father’s job at birth (ISCO-68) and TGCT in offspring was observed (OR = 1.98, CI 1.18–3.30). Specific jobs as “Protective service workers (5–8)” (OR = 2.40, CI 1.20–4.81) and “Transport equipment operators (9–8)” (OR = 1.96, CI 1.14–3.37) showed positive associations (Table 2).

**Table 2:** Odds ratios (OR) and 95% confidence intervals (CI) for TGCT associated with father’s job (ISCO-1968) and industry sector (NAF-1999) at birth, overall, case–control study, *N* = 1,124, France, 2015–2018.

	N cases/ *N* controls^1,3^	Crude OR (95% CI)	*N* cases/ *N* controls^2,4^	Adjusted OR^5^ (95% CI)
ISCO-1968 codes				
**Professional, technical, and related workers (0/1)**	89/175	0.78 (0.58–1.05)	88/173	0.74 (0.54–1.00)
Architects, engineers, and related technicians (0–2/0–3)	31/58	0.89 (0.56–1.42)	31/57	0.86 (0.53–1.39)
Draughtsmen (0–32)	7/14	0.67 (0.26–1.72)	7/14	0.69 (0.26–1.83)
Electrical and electronics engineering (0–34)	5/7	1.19 (0.36–3.87)	5/7	0.82 (0.24–2.83)
Medical, dental, veterinary, and related workers (0–6/0–7)	11/33	0.48 (0.24–0.97)	11/33	0.51 (0.25–1.05)
Medical doctors (0–61)	10/20	0.69 (0.31–1.51)	10/20	0.73 (0.33–1.63)
Specialized physician (0–61.20)	5/8	0.86 (0.26–2.83)	5/8	1.03 (0.31–3.47)
Statisticians and related technicians (0–8)	10/12	1.07 (0.44–2.59)	10/12	1.04 (0.42–2.58)
Systems analysts (0–83)	8/8	1.11 (0.39–3.17)	8/8	1.17 (0.40–3.46)
Systems analyst (0–83.10)	8/8	1.25 (0.44–3.60)	8/8	1.33 (0.44–3.98)
Accountants (1–1)	7/12	1.03 (0.40–2.67)	7/11	1.23 (0.46–3.29)
Accountants (110)	7/12	0.99 (0.38–2.57)	7/11	1.18 (0.45–3.14)
Teachers (1–3)	14/29	0.82 (0.42–1.58)	13/29	0.66 (0.32–1.35)
Secondary education teachers (1–32)	8/11	1.14 (0.45–2.90)	8/11	0.93 (0.34–2.52)
**Administrative and managerial workers (2)**	21/41	0.75 (0.43–1.30)	21/41	0.74 (0.42–1.31)
Managers (2–1)	18/41	0.63 (0.35–1.12)	18/41	0.61 (0.34–1.11)
General managers (2–11)	5/8	1.00 (0.32–3.11)	5/8	1.02 (0.31–3.36)
General manager (2–11.10)	5/8	1.02 (0.33–3.18)	5/8	1.08 (0.33–3.55)
Managers not elsewhere classified (2–19)	10/23	0.60 (0.28–1.30)	10/23	0.60 (0.27–1.32)
Other managers (2–19.90)	5/15	0.47 (0.16–1.32)	5/15	0.40 (0.13–1.18)
**Clerical and related workers (3)**	36/53	1.10 (0.70–1.73)	36/52	1.14 (0.71–1.82)
Bookkeepers, cashiers, and related workers (3–3)	11/12	1.55 (0.66–3.64)	11/11	1.42 (0.58–3.49)
Bookkeepers and cashiers (3–31)	5/5	1.65 (0.47–5.81)	5/5	1.42 (0.39–5.11)
Bookkeepers, cashiers, and related workers not elsewhere classified (3–39)	6/7	1.44 (0.46–4.56)	6/6	1.40 (0.40–4.88)
Finance clerk (3–39.40)	6/7	1.38 (0.43–4.38)	6/6	1.27 (0.37–4.41)
Mail distribution clerks (3–7)	5/5	1.84 (0.53–6.42)	5/5	1.98 (0.53–7.38)
Mail distribution clerks (3–70)	5/5	1.81 (0.52–6.29)	5/5	1.92 (0.52–7.12)
Clerical and related workers not elsewhere classified (3–9)	14/27	0.80 (0.41–1.55)	14/27	0.86 (0.44–1.69)
Stock clerks (3–91)	5/14	0.51 (0.18–1.45)	5/14	0.60 (0.21–1.71)
Correspondence and reporting clerks (3–93)	8/11	1.17 (0.46–2.96)	8/11	1.18 (0.46–3.03)
Office clerk (general) (3–93.10)	8/10	1.31 (0.50–3.39)	8/10	1.27 (0.48–3.37)
**Sales workers (4)**	35/42	1.28 (0.79–2.08)	35/40	1.47 (0.89–2.42)
Working proprietors (wholesale and retail trade) (4–1)	7/8	1.47 (0.52–4.17)	7/8	1.71 (0.59–4.92)
Working proprietors (wholesale and retail trade) (4–10)	7/7	1.61 (0.55–4.71)	7/7	1.86 (0.63–5.52)
Working proprietor (retail trade) (4–10.30)	7/6	1.73 (0.56–5.40)	7/6	1.96 (0.62–6.18)
Technical salesmen, commercial travelers, and manufacturers’ agents (4–3)	16/19	1.26 (0.62–2.57)	16/18	1.48 (0.71–3.09)
Technical salesmen and service advisers (4–31)	14/16	1.25 (0.59–2.67)	14/15	1.47 (0.67–3.22)
Technical salesman (4–31.20)	14/16	1.18 (0.55–2.55)	14/15	1.42 (0.64–3.15)
Salesmen, shop assistants, and related workers (4–5)	6/8	1.06 (0.35–3.16)	6/8	1.08 (0.35–3.32)
**Service workers (5)**	38/33	**2.00 (1.21**–**3.30)**	38/35	**1.98 (1.18**–**3.30)**
Cooks, waiters, bartenders, and related workers (5–3)	7/8	1.32 (0.47–3.74)	7/8	1.21 (0.42–3.50)
Protective service workers (5–8)	22/15	**2.46 (1.24**–**4.86)**	22/15	**2.40 (1.20**–**4.81)**
Protective service workers not elsewhere classified (5–89)	11/9	1.93 (0.78–4.79)	11/9	1.74 (0.69–4.36)
Other protective service workers (5–89.90)	8/9	1.43 (0.53–3.82)	8/9	1.25 (0.46–3.41)
**Agricultural, animal husbandry and forestry workers, fishermen, and hunters (6)**	30/37	1.16 (0.70–1.93)	29/37	1.12 (0.66–1.90)
Farmers (6–1)	22/21	1.61 (0.86–3.02)	21/21	1.62 (0.85–3.10)
General farmers (6–11)	11/12	1.17 (0.50–2.75)	10/12	1.04 (0.42–2.57)
General farmer (6–11.10)	11/11	1.22 (0.51–2.96)	10/11	1.11 (0.44–2.80)
Specialized farmers (6–12)	11/8	2.36 (0.93–6.01)	11/8	**2.66 (1.03**–**6.90)**
Agricultural and animal husbandry workers (6–2)	6/10	0.83 (0.29–2.37)	6/10	0.82 (0.28–2.39)
**Production and related workers, transport equipment operators, and laborers (7/8/9)**	155/234	1.01 (0.77–1.32)	154/233	1.01 (0.77–1.33)
Production supervisors and general foremen (7–0)	7/12	0.91 (0.35–2.36)	7/12	1.03 (0.39–2.73)
Production supervisors and general foremen (7–00)	7/12	0.88 (0.34–2.28)	7/12	0.98 (0.37–2.59)
Food and beverage processors (7–7)	8/16	0.78 (0.32–1.87)	8/16	0.74 (0.30–1.82)
Blacksmiths, toolmakers, and machine-tool operators (8–3)	14/13	1.55 (0.71–3.37)	14/13	1.44 (0.64–3.24)
Machinery fitters, machine assemblers, and precision-instrument makers [except electrical] (8–4)	21/33	0.92 (0.52–1.62)	20/33	0.84 (0.46–1.51)
Motor vehicle mechanics (8–43)	8/10	1.12 (0.43–2.91)	8/10	1.02 (0.39–2.69)
Automobile mechanic (8–43.20)	6/6	1.50 (0.47–4.73)	6/6	1.36 (0.42–4.35)
Machinery fitters, machine assemblers, and precision-instrument makers [except electrical] not elsewhere classified (8–49)	8/15	0.73 (0.30–1.76)	8/15	0.75 (0.31–1.82)
Electrical fitters and related electrical and electronics workers (8–5)	9/21	0.62 (0.28–1.40)	9/21	0.52 (0.23–1.19)
Electrical wiremen (8–55)	5/8	0.95 (0.30–2.96)	5/8	0.78 (0.25–2.50)
Plumbers, welders, sheet-metal and structural metal preparers, and erectors (8–7)	14/21	1.02 (0.51–2.07)	14/21	1.05 (0.51–2.14)
Bricklayers, carpenters, and other construction workers (9–5)	19/37	0.75 (0.42–1.33)	19/37	0.84 (0.47–1.52)
Bricklayers, stonemasons, and tile setters (9–51)	14/13	1.40 (0.65–3.05)	14/13	1.59 (0.72–3.52)
Material handling and related equipment operators, dockers, and freight handlers (9–7)	5/19	0.42 (0.16–1.15)	5/19	**0.33 (0.12**–**0.95)**
Transport equipment operators (9–8)	34/30	**1.77 (1.06**–**2.98)**	34/29	**1.96 (1.14**–**3.37)**
Motor vehicle drivers (9–85)	30/28	1.57 (0.91–2.70)	30/27	1.67 (0.95–2.92)
Lorry and van driver (local transport) (9–85.50)	12/11	1.49 (0.63–3.53)	12/11	1.28 (0.52–3.14)
Lorry and van driver (long-distance transport) (9–85.60)	5/9	0.84 (0.27–2.62)	5/8	0.98 (0.29–3.31)
NAF-1999 codes				
**Agriculture, hunting, and forestry (01, 02)**	31/38	1.20 (0.73–1.99)	30/38	1.21 (0.72–2.03)
Agriculture, hunting, and related service activities (01)	28/32	1.33 (0.78–2.28)	27/32	1.38 (0.80–2.40)
Growing of crops combined with farming of animals (mixed farming) (01.3)	11/12	1.13 (0.48–2.66)	10/12	1.02 (0.41–2.54)
Growing of crops combined with farming of animals (mixed farming) (01.3Z)	11/12	1.23 (0.52–2.93)	10/12	1.13 (0.45–2.82)
**Manufacturing (15–37)**	61/105	0.86 (0.61–1.23)	61/103	0.85 (0.59–1.22)
Food industry (15)	7/14	0.70 (0.27–1.79)	7/13	0.73 (0.27–1.95)
Publishing, printing, reproduction (22)	8/5	3.05 (0.97–9.58)	8/5	**3.53 (1.10**–**11.30)**
Metalworking (28)	18/23	1.14 (0.60–2.17)	18/23	1.13 (0.58–2.19)
Manufacture of other transport equipment (35)	5/6	1.23 (0.37–4.15)	5/6	1.03 (0.30–3.60)
**Electricity, gas, and water supply (40, 41)**	5/5	1.46 (0.41–5.18)	5/5	1.61 (0.44–5.89)
**Construction (45)**	44/69	0.93 (0.62–1.41)	44/69	0.98 (0.64–1.50)
Construction of building or civil engineering works (45.2)	15/23	0.96 (0.49–1.89)	15/23	1.15 (0.57–2.31)
Installation works (45.3)	9/17	0.79 (0.34–1.84)	9/17	0.72 (0.31–1.71)
Building completion work (45.4)	12/13	1.21 (0.53–2.76)	12/13	1.28 (0.55–2.95)
**Wholesale and retail trade, repair of motor vehicles, motorcycles, and personal and household goods (50, 51, 52)**	48/62	1.23 (0.81–1.85)	47/62	1.17 (0.76–1.79)
Sale and repair of motor vehicles (50)	14/13	1.64 (0.75–3.58)	14/13	1.46 (0.66–3.24)
Maintenance and repair services of motor vehicles (50.2)	12/11	1.67 (0.71–3.88)	12/11	1.55 (0.65–3.67)
Maintenance and repair services of motor vehicles (50.2Z)	12/11	1.67 (0.71–3.89)	12/11	1.54 (0.65–3.65)
Wholesale trade and trade intermediaries (51)	8/13	0.85 (0.34–2.14)	8/13	0.92 (0.36–2.35)
Retail and repair of household goods (52)	26/36	1.18 (0.70–2.01)	25/36	1.12 (0.64–1.93)
Food retailing in specialized stores (52.2)	7/10	1.11 (0.41–3.00)	7/10	1.15 (0.42–3.14)
Other retail in specialized stores (52.4)	12/12	1.62 (0.71–3.70)	11/12	1.41 (0.60–3.35)
**Hotels and restaurants (55)**	10/13	1.26 (0.54–2.95)	10/13	1.18 (0.49–2.84)
Restaurants (55.3)	5/9	0.85 (0.28–2.63)	5/9	0.85 (0.27–2.69)
**Transport, storage, and communication (60, 61, 62, 63, 64)**	42/60	1.16 (0.76–1.77)	42/59	1.16 (0.75–1.80)
Land transport (60)	29/35	1.31 (0.78–2.22)	29/34	1.41 (0.82–2.41)
Transport via railways (60.1)	6/9	1.14 (0.39–3.31)	6/9	1.38 (0.47–4.11)
Transport via railways (60.1Z)	6/9	1.13 (0.39–3.29)	6/9	1.34 (0.45–4.00)
Urban and road transport (60.2)	22/25	1.37 (0.75–2.50)	22/24	1.41 (0.75–2.64)
Local road transport of goods (60.2 L)	8/8	1.55 (0.56–4.31)	8/8	1.35 (0.47–3.88)
Post and telecommunications (64)	8/19	0.76 (0.33–1.78)	8/19	0.61 (0.25–1.50)
Post and courier activities (64.1)	5/8	1.07 (0.34–3.39)	5/8	0.93 (0.28–3.07)
National post activities (64.1A)	5/8	1.10 (0.35–3.48)	5/8	0.96 (0.29–3.19)
**Financial intermediation (65, 66, 67)**	11/19	0.97 (0.45–2.10)	11/17	0.89 (0.40–2.01)
Financial intermediation (65)	10/15	1.13 (0.49–2.59)	10/14	1.00 (0.42–2.37)
Monetary intermediation (65.1)	10/15	1.10 (0.48–2.52)	10/14	0.96 (0.40–2.30)
**Real estate, renting, and business activities (70, 71, 72, 73, 74)**	21/43	0.79 (0.45–1.38)	21/42	0.81 (0.46–1.44)
Services provided primarily to businesses (74)	15/24	1.04 (0.53–2.03)	15/23	1.07 (0.53–2.15)
Architectural and engineering activities (74.2)	6/19	0.47 (0.18–1.21)	6//19	0.44 (0.16–1.19)
Engineering, technical studies (74.2C)	5/14	0.53 (0.19–1.52)	5/14	0.53 (0.18–1.60)
**Public administration and defence; compulsory social security (75)**	40/45	1.49 (0.95–2.34)	40/45	1.54 (0.97–2.45)
General, economic, and social administration (75.1)	16/14	1.98 (0.95–4.14)	16/14	2.02 (0.96–4.25)
General public administration (75.1A)	12/13	1.57 (0.70–3.53)	12/13	1.64 (0.72–3.74)
Public prerogative services (75.2)	24/31	1.20 (0.68–2.11)	24/31	1.23 (0.69–2.20)
Defence (75.2C)	13/24	0.79 (0.39–1.60)	13/24	0.76 (0.37–1.58)
**Education (80)**	17/31	0.96 (0.52–1.78)	16/31	0.81 (0.42–1.58)
Secondary education (80.2)	10/11	1.38 (0.57–3.35)	10/11	1.15 (0.45–2.94)
**Health and social work (85)**	18/41	0.66 (0.37–1.18)	18/41	0.68 (0.37–1.25)
Activities for human health (85.1)	16/32	0.72 (0.38–1.35)	16/32	0.74 (0.38–1.42)
Hospital activities (85.1A)	7/12	1.00 (0.38–2.64)	7/12	1.10 (0.40–3.01)
Medical practice (85.1C)	6/13	0.61 (0.22–1.67)	6/13	0.62 (0.22–1.73)
**Other community, social, and personal service activities (90, 91, 92, 93)**	7/13	1.00 (0.39–2.56)	7/13	1.07 (0.41–2.79)

Significant differences in bold.^1^For ISCO-1968 codes, in total: 416/647 for one and two digits, 408/620 for three digits, and 365/561 for four and five digits in crude analyses.

^2^For ISCO-1968 codes, in total: 413/641 for one and two digits, 405/614 for three digits, and 362/556 for four and five digits in adjusted analyses.

^3^For NAF-1999 codes, in total: 376/592 for two digits, 342/530 for three digits, and 288/455 for four digits in crude analyses.

^4^For NAF-1999, in total: 379/598 for two digits, 345/533 for three digits, and 290/456 for four digits in adjusted analyses.

^5^Adjusted for sibship size, born from multiple pregnancies, personal history of testicular trauma, family history of testicular cancer, and family history of cryptorchidism.

No heterogeneity between the two TGCT subtypes was observed (Table 3). For seminoma, positive associations were found for “Service workers (5)” (OR = 1.94, CI 1.03–3.67; p-het 0.86), “Protective service workers (5–8)” (OR = 2.56, CI 1.12–5.83; p-het 0.50), “Specialized farmers (6–12)” (OR = 3.02, CI 1.05–9.33; p-het 0.77), and “Transport equipment operators (9–8)” (OR = 2.07, CI 1.08–3.94; p-het 0.79). No significant associations were seen with non-seminomas.

**Table 3:** Odds ratios (OR) and 95% confidence intervals (CI) for TGCT associated with father’s job (ISCO-1968) and industry sector (NAF-1999) at birth, according to TGCT histological subtypes, case–control study, *N* = 1,124, France, 2015–2018.

	Non-seminomas			Seminomas			P-HET**
	*N* cases/ *N* controls	Crude OR (95% CI)	Adjusted OR* (95% CI)	*N* cases/ *N* controls	Crude OR (95% CI)	Adjusted OR* (95% CI)	
ISCO-1968 codes							
**Professional, technical, and related workers (0/1)**	36/173	0.79 (0.51–1.21)	0.75 (0.48–1.17)	44/173	0.82 (0.56–1.21)	0.82 (0.55–1.22)	0.77
Architects, engineers, and related technicians (0–2/0–3)	6/30	0.78 (0.30–2.00)	0.65 (0.24–1.76)	11/30	1.26 (0.60–2.61)	1.23 (0.58–2.60)	0.30
Teachers (1–3)	5/29	0.82 (0.30–2.27)	0.78 (0.27–2.23)	8/29	1.04 (0.48–2.27)	0.87 (0.37–2.05)	0.88
**Administration and managerial workers (2)**	9/41	0.73 (0.33–1.62)	0.77 (0.34–1.73)	9/41	0.71 (0.33–1.51)	0.65 (0.30–1.42)	0.77
Managers (2–1)	7/41	0.54 (0.22–1.29)	0.56 (0.23–1.37)	8/41	0.62 (0.28–1.37)	0.56 (0.25–1.27)	0.99
**Clerical and related workers (3)**	11/52	0.84 (0.42–1.69)	0.94 (0.46–1.92)	19/52	1.15 (0.65–2.04)	1.20 (0.67–2.15)	0.60
Clerical and related workers not elsewhere classified (3–9)	6/27	0.89 (0.35–2.25)	0.93 (0.36–2.36)	5/27	0.56 (0.21–1.51)	0.61 (0.23–1.67)	0.55
**Sales workers (4)**	14/40	1.23 (0.62–2.41)	1.37 (0.68–2.73)	16/40	1.18 (0.64–2.18)	1.29 (0.68–2.43)	0.90
Technical salesmen, commercial travelers, and manufacturers’ agents (4–3)	5/18	0.97 (0.34–2.80)	1.06 (0.36–3.13)	6/18	1.00 (0.38–2.61)	1.10 (0.41–2.95)	0.96
Technical salesmen and service advisers (4–31)	5/15	1.07 (0.37–3.09)	1.16 (0.38–3.47)	5/15	0.95 (0.34–2.67)	1.05 (0.36–3.05)	0.91
Technical salesman (4–31.20)	5/15	1.05 (0.36–3.07)	1.14 (0.38–3.46)	5/15	0.90 (0.32–2.53)	1.01 (0.35–2.95)	0.88
**Service workers (5)**	15/33	1.78 (0.91–3.50)	1.79 (0.89–3.57)	19/33	**1.99 (1.08**–**3.68)**	**1.94 (1.03**–**3.67)**	0.86
Protective service workers (5–8)	7/15	1.57 (0.61–4.03)	1.67 (0.64–4.32)	12/15	**2.71 (1.21**–**6.06)**	**2.56 (1.12**–**5.83)**	0.50
**Agricultural, animal husbandry and forestry workers, fishermen, and hunters (6)**	15/37	1.39 (0.72–1.53)	1.22 (0.61–2.41)	14/37	1.22 (0.64–2.32)	1.18 (0.61–2.28)	0.95
Farmers (6–1)	9/21	1.54 (0.66–3.57)	1.43 (0.59–3.46)	12/21	2.01 (0.95–4.25)	2.00 (0.93–4.32)	0.57
Specialized farmers (6–12)	5/8	2.17 (0.65–7.25)	2.45 (0.74–8.11)	6/8	**3.02 (1.02**–**8.92)**	**3.12 (1.05**–**9.33)**	0.77
**Production and related workers, transport equipment operators, and laborers (7/8/9)**	65/233	1.05 (0.72–1.53)	1.05 (0.72–1.55)	73/233	0.92 (0.65–1.31)	0.93 (0.65–1.33)	0.64
Blacksmiths, toolmakers, and machine tool operators (8–3)	5/13	1.39 (0.46–4.22)	1.30 (0.42–4.04)	8/13	1.82 (0.72–4.59)	1.51 (0.57–4.04)	0.84
Plumbers, welders, sheet metal and structural metal preparers, and erectors (8–7)	6/21	1.14 (0.44–2.99)	1.11 (0.42–2.96)	5/21	0.61 (0.23–1.67)	0.62 (0.22–1.72)	0.42
Bricklayers, carpenters, and other construction workers (9–5)	7/37	0.65 (0.27–1.56)	0.66 (0.27–1.61)	10/37	0.80 (0.39–1.67)	0.92 (0.44–1.94)	0.57
Transport equipment operators (9–8)	13/29	1.70 (0.83–3.49)	1.81 (0.87–3.79)	19/29	**1.95 (1.05**–**3.64)**	**2.07 (1.08**–**3.94)**	0.79
Bricklayers, stonemasons, and tile setters (9–51)	7/13	1.49 (0.55–4.04)	1.43 (0.51–4.00)	6/13	1.44 (0.53–3.90)	1.60 (0.58–4.41)	0.87
Motor vehicle drivers (9–85)	11/27	1.40 (0.65–3.00)	1.47 (0.67–3.20)	17/27	1.81 (0.95–3.48)	1.85 (0.94–3.62)	0.66
NAF-1999 codes							
**Agriculture, hunting, and forestry (01, 02)**	14/38	1.41 (0.72–2.76)	1.27 (0.63–2.56)	16/38	1.30 (0.70–2.42)	1.27 (0.67–2.39)	1.00
Agriculture, hunting, and related service activities (01)	13/32	1.68 (0.82–3.42)	1.62 (0.78–3.37)	14/32	1.37 (0.70–2.68)	1.35 (0.68–2.67)	0.73
**Manufacturing (15–37)**	23/103	0.77 (0.46–1.29)	0.75 (0.45–1.27)	33/103	0.91 (0.58–1.41)	0.87 (0.55–1.37)	0.68
**Construction (45)**	19/69	0.92 (0.51–1.65)	0.95 (0.52–1.73)	19/69	0.83 (0.48–1.44)	0.94 (0.53–1.65)	0.98
Building of complete constructions or parts thereof; civil engineering (45.2)	5/23	0.72 (0.26–2.03)	0.80 (0.28–2.32)	8/23	1.04 (0.44–2.45)	1.16 (0.48–2.79)	0.60
Building completion (45.4)	5/13	1.23 (0.40–3.75)	1.21 (0.39–3.76)	6/13	1.12 (0.40–3.08)	1.35 (0.48–3.77)	0.89
**Wholesale and retail trade, repair of motor vehicles, motorcycles, and personal and household goods (50, 51, 52)**	19/62	1.19 (0.67–2.10)	1.21 (0.67–2.18)	21/62	0.99 (0.58–1.69)	0.99 (0.57–1.71)	0.62
Sale, maintenance, and repair of motor vehicles and motorcycles; retail sale of automotive fuel (50)	5/13	1.52 (0.51–4.58)	1.62 (0.53–4.93)	5/13	0.98 (0.33–2.88)	0.92 (0.30–2.84)	0.48
Retail and repair of household goods (52)	11/36	1.29 (0.63–2.65)	1.24 (0.59–2.62)	12/36	0.97 (0.49–1.91)	0.97 (0.49–1.92)	0.63
**Transport, storage, and communication (60, 61, 62, 63, 64)**	17/59	1.15 (0.64–2.08)	1.14 (0.63–2.08)	22/59	1.15 (0.67–1.96)	1.13 (0.66–1.95)	0.99
Land transport (60)	11/34	1.27 (0.61–2.65)	1.37 (0.65–2.89)	16/34	1.31 (0.69–2.47)	1.35 (0.70–2.60)	0.97
Other land transport (60.2)	7/24	1.11 (0.46–2.70)	1.12 (0.45–2.80)	13/24	1.57 (0.76–3.25)	1.59 (0.75–3.38)	0.56
**Real estate, renting, and business activities (70, 71, 72, 73, 74)**	10/42	0.93 (0.43–2.02)	0.89 (0.40–1.98)	8/42	0.59 (0.27–1.29)	0.63 (0.28–1.41)	0.55
Services provided primarily to businesses (74)	7/23	1.11 (0.44–2.77)	1.07 (0.41–2.76)	6/23	0.79 (0.31–1.98)	0.89 (0.35–2.30)	0.79
**Public administration and defence; compulsory social security (75)**	19/45	1.93 (1.06–3.53)	1.99 (1.09–3.65)	16/45	1.07 (0.58–1.96)	1.10 (0.59–2.03)	0.18
General, economic, and social administration (75.1)	8/14	3.24 (1.25–8.40)	3.21 (1.23–8.39)	6/14	1.23 (0.46–3.32)	1.36 (0.50–3.66)	0.22
Public prerogative services (75.2)	11/31	1.35 (0.63–2.89)	1.39 (0.64–2.99)	10/31	0.96 (0.45–2.04)	0.91 (0.42–1.99)	0.45
Defence (75.2C)	8/24	1.14 (0.47–2.75)	1.11 (0.45–2.73)	5/24	0.62 (0.23–1.70)	0.57 (0.20–1.62)	0.34
**Education (80)**	6/31	0.98 (0.39–2.50)	0.95 (0.36–2.49)	10/31	1.21 (0.58–2.50)	1.04 (0.47–2.30)	0.89
**Health and social work (85)**	6/41	0.60 (0.24–1.49)	0.61 (0.24–1.53)	10/41	0.74 (0.35–1.56)	0.72 (0.34–1.54)	0.78
Human health activities (85.1)	5/32	0.65 (0.24–1.78)	0.67 (0.24–1.85)	10/32	0.89 (0.41–1.90)	0.86 (0.39–1.89)	0.70

Significant differences in bold.*Adjusted for sibship size, born from multiple pregnancies, personal history of testicular trauma, family history of testicular cancer, and family history of cryptorchidism.

**p-value for heterogeneity derived from the likelihood ratio test, comparing seminoma versus non-seminoma tumours in adjusted models.

Few significant associations between father’s industry at birth (NAF-1999) and TGCT in adulthood were observed with any of the main job categories (Table 2). Statistically significant increased OR was only observed for “Publishing, printing, and reproduction (22)” after adjustment (OR = 3.53, CI 1.10–11.30). While no significant association was observed for seminomas, statistically significant increases were observed for non-seminomas and “Public administration and defence; compulsory social security (57)” industry (OR = 1.99, CI 1.09–3.65; p-het 0.18), and “General, economic, and social administration (75.1)” (OR = 3.21, CI 1.23–8.39; p-het 0.22) (Table 3).

### 3.3 TGCT risk and mother’s employment

Few significant associations with TGCT in offspring were seen with the main mothers’ job categories (ISCO-68) (Table 4). We observed a positive association for “Secondary education teachers (1–32)” (OR = 2.27, CI 1.09–4.76). No significant association was observed between mother’s job at birth (ISCO-68) and seminomas (Table 5). A statistically significant increase in non-seminoma risk was observed for “Secondary education teacher (1–32)” (OR = 4.67, CI 1.87–11.67; p-het 0.09).

**Table 4:** Odds ratios (OR) and 95% confidence intervals (CI) for TGCT associated with mother’s job (ISCO-1968) and industry sector (NAF-1999) at birth, overall, case–control study, *N* = 1,124, France, 2015–2018.

	*N* cases/ *N* controls	Crude OR (95% CI)	*N* cases/ *N* controls	Adjusted OR* (95% CI)
ISCO-1968 codes				
**Professional, technical, and related workers (0/1)**	86/137	0.98 (0.72–1.34)	85/137	0.95 (0.69–1.31)
Medical, dental, veterinary, and related workers (0–6/0–7)	24/50	0.71 (0.43–1.19)	23/50	0.43 (0.05–4.01)
Medical doctors (0–61)	5/7	1.03 (0.31–3.41)	5/7	1.16 (0.34–3.92)
Professional nurses (0–71)	14/24	0.92 (0.46–1.81)	13/24	0.87 (0.43–1.77)
Professional nurse (general) (0–71.10)	6/15	0.65 (0.25–1.71)	6/15	0.72 (0.27–1.92)
Accountants (1–1)	14/13	1.95 (0.90–4.23)	14/13	1.89 (0.87–4.12)
Accountants (1–10)	14/13	1.95 (0.90–4.23)	14/13	1.88 (0.86–4.11)
Auditor (1–10.20)	14/11	**2.30 (1.02**–**5.18)**	14/11	2.26 (1.00–5.11)
Teachers (1–3)	32/41	1.18 (0.72–1.93)	32/41	1.17 (0.70–1.94)
Secondary education teachers (1–32)	19/14	**2.30 (1.12**–**4.71)**	19/14	**2.27 (1.09**–**4.76)**
Primary education teachers (1–33)	10/19	0.76 (0.35–1.67)	10/19	0.75 (0.33–1.70)
First-level education teacher (1–33.20)	10/19	0.79 (0.36–1.74)	10/19	0.77 (0.34–1.77)
Professional, technical, and related workers not elsewhere classified (1–9)	7/17	0.73 (0.30–1.80)	7/17	0.69 (0.27–1.75)
Social workers (1–93)	5/7	1.36 (0.42–4.44)	5/7	1.10 (0.32–3.72)
**Administrative and managerial workers (2)**	6/11	0.73 (0.25–2.09)	6/11	0.68 (0.23–1.97)
Managers (2–1)	6/11	0.73 (0.25–2.09)	6/11	0.68 (0.23–1.97)
Managers not elsewhere classified (2–19)	6/9	0.83 (0.28–2.50)	6/9	0.80 (0.26–2.43)
**Clerical and related workers (3)**	94/125	1.19 (0.88–1.62)	93/123	1.16 (0.84–1.61)
Stenographers, typists, and card-and tape-punching machine operators (3–2)	28/45	1.00 (0.61–1.64)	28/45	0.94 (0.57–1.57)
Stenographers, typists, and teletypists (3–21)	28/45	1.00 (0.61–1.64)	28/45	0.94 (0.57–1.58)
Stenographer-typist (general) (3–21.10)	18/32	0.86 (0.47–1.58)	18/32	0.83 (0.44–1.54)
Stenographic secretary (3–21.20)	10/13	1.36 (0.59–3.15)	10/13	1.27 (0.53–3.02)
Bookkeepers, cashiers, and related workers (3–3)	7/19	0.52 (0.21–1.26)	7/18	0.55 (0.22–1.37)
Bookkeepers and cashiers (3–31)	6/13	0.63 (0.23–1.70)	6/12	0.71 (0.25–2.01)
Clerical and related workers not elsewhere classified (3–9)	45/49	1.38 (0.90–2.12)	44/49	1.30 (0.83–2.04)
Correspondence and reporting clerks (3–93)	37/41	1.42 (0.89–2.27)	37/41	1.35 (0.83–2.20)
Office clerk (general) (3–93.10)	32/35	1.51 (0.91–2.52)	32/35	1.43 (0.84–2.44)
**Sales workers (4)**	23/35	1.04 (0.60–1.81)	23/35	1.04 (0.59–1.83)
Salesmen, shop assistants, and related workers (4–5)	14/19	1.15 (0.56–2.37)	14/19	1.11 (0.53–2.31)
Salesmen, shop assistants, and demonstrators (4–51)	14/18	1.20 (0.58–2.48)	14/18	1.14 (0.54–2.41)
Retail trade salesman (4–51.30)	9/15	0.86 (0.36–2.05)	9/15	0.88 (0.36–2.11)
**Service workers (5)**	46/75	0.89 (0.60–1.32)	46/73	0.92 (0.61–1.39)
Cooks, waiters, bartenders, and related workers (5–3)	6/12	0.60 (0.22–1.70)	6/12	0.74 (0.26–2.11)
Maids and related housekeeping service workers not elsewhere classified (5–4)	8/6	1.76 (0.59–5.24)	8/6	1.71 (0.56–5.27)
Maids and related housekeeping service workers not elsewhere classified (5–40)	8/6	1.74 (0.58–5.21)	8/6	1.69 (0.55–5.21)
Building caretakers, charworkers, cleaners, and related workers (5–5)	12/21	0.77 (0.37–1.60)	12/21	0.72 (0.34–1.52)
Charworkers, cleaners, and related workers (5–52)	12/20	0.80 (0.38–1.67)	12/20	0.77 (0.36–1.63)
Charworker (5–52.20)	12/20	0.82 (0.39–1.71)	12/20	0.78 (0.36–1.66)
Service workers not elsewhere classified (5–9)	13/22	1.06 (0.52–2.14)	13/20	1.17 (0.56–2.45)
Other service workers (5–99)	13/22	1.06 (0.52–2.13)	13/20	1.18 (0.56–2.45)
Nursing aid (5–99.40)	12/21	1.04 (0.50–2.16)	12/19	1.15 (0.54–2.46)
**Agricultural, animal husbandry, and forestry workers, fishermen, and hunters (6)**	10/14	1.01 (0.43–2.32)	9/14	0.96 (0.40–2.33)
Farmers (6–1)	9/10	1.31 (0.52–3.29)	8/10	1.23 (0.46–3.28)
Specialized farmers (6–12)	6/7	1.17 (0.38–3.57)	5/7	1.06 (0.31–3.60)
**Production and related workers, transport equipment operators, and laborers (7/8/9)**	19/46	0.59 (0.34–1.04)	19/44	0.62 (0.35–1.12)
Tailors, dressmakers, sewers, upholsterers, and related workers (7–9)	5/12	0.56 (0.19–1.65)	5/12	0.60 (0.20–1.81)
NAF-1999 codes				
**Agriculture, hunting, and forestry (01, 02)**	10/14	0.96 (0.41–2.24)	9/14	0.90 (0.37–2.20)
Agriculture, hunting, and related service activities (01)	10/14	0.96 (0.41–2.24)	9/14	0.90 (0.37–2.20)
Farming (01.2)	5/6	1.09 (0.32–3.67)	–	–
**Manufacturing (15–37)**	21/43	0.70 (0.40–1.22)	21/43	0.70 (0.40–1.24)
Food industry (15)	5/8	0.80 (0.25–2.58)	5/8	0.77 (0.23–2.59)
Clothing and fur industry (18)	6/10	0.83 (0.29–2.38)	6/10	0.88 (0.30–2.59)
Manufacture of textile clothing (18.2)	6/10	0.87 (0.30–2.47)	6/10	0.93 (0.32–2.72)
**Wholesale and retail trade, repair of motor vehicles, motorcycles, and personal and household goods (50, 51, 52)**	25/44	0.89 (0.53–1.49)	25/43	0.91 (0.53–1.55)
Retail and repair of household goods (52)	22/40	0.83 (0.48–1.44)	22/39	0.89 (0.51–1.56)
Retail trade in non-specialized stores (52.1)	5/10	0.75 (0.24–2.29)	5/9	0.89 (0.28–2.89)
Supermarkets (52.1D)	5/9	0.79 (0.24–2.52)	5/8	0.97 (0.28–3.39)
Other retail in specialized stores (52.4)	8/11	1.04 (0.40–2.67)	8/11	1.01 (0.39–2.62)
**Hotels and restaurants (55)**	10/19	0.66 (0.30–1.47)	10/19	0.71 (0.31–1.61)
Restaurants (55.3)	7/12	0.79 (0.30–2.07)	7/12	0.88 (0.32–2.39)
**Transport, storage, and communication (60, 61, 62, 63, 64)**	9/14	0.92 (0.38–2.20)	9/13	0.93 (0.38–2.31)
**Financial intermediation (65, 66, 67)**	6/15	0.60 (0.23–1.58)	6/15	0.59 (0.22–1.56)
**Real estate, renting, and business activities (70, 71, 72, 73, 74)**	15/19	1.21 (0.60–2.45)	15/18	1.17 (0.56–2.45)
Services provided primarily to businesses (74)	9/11	1.27 (0.51–3.18)	9/11	1.09 (0.42–2.82)
Legal, accounting, and management consulting activities (74.1)	7/7	1.71 (0.58–5.02)	7/7	1.40 (0.45–4.33)
**Public administration and defence; compulsory social security (75)**	18/34	0.77 (0.42–1.41)	18/33	0.94 (0.50–1.76)
General, economic, and social administration (75.1)	11/23	0.77 (0.36–1.62)	11/22	0.90 (0.41–1.98)
General public administration (75.1A)	9/21	0.67 (0.29–1.51)	9/20	0.75 (0.32–1.78)
**Education (80)**	37/47	1.16 (0.73–1.84)	37/47	1.17 (0.73–1.89)
Primary education (80.1)	14/22	0.85 (0.43–1.71)	14/22	0.83 (0.41–1.71)
Primary education (80.1Z)	14/22	0.78 (0.39–1.59)	14/22	0.77 (0.37–1.60)
Secondary education (80.2)	20/14	**2.43 (1.19**–**4.95)**	20/14	**2.35 (1.13**–**4.88)**
General secondary education (80.2A)	10/6	2.22 (0.76–6.48)	10/6	1.99 (0.67–5.95)
**Health and social work (85)**	54/93	0.89 (0.62–1.29)	52/91	0.85 (0.58–1.25)
Activities for human health (85.1)	27/64	0.65 (0.41–1.05)	27/62	0.68 (0.42–1.11)
Hospital activities (85.1A)	20/37	0.83 (0.47–1.49)	20/36	0.84 (0.46–1.52)
Social action (85.3)	12/16	1.12 (0.51–2.45)	12/16	1.09 (0.48–2.44)
**Other community, social, and personal services activities (90, 91, 92, 93)**	9/14	0.97 (0.41–2.31)	9/14	1.01 (0.41–2.46)
Recreational, cultural and sporting activities (92)	5/6	1.14 (0.33–3.93)	5/6	1.14 (0.33–3.93)
**Private households with employed persons (95)**	5/10	0.67 (0.22–2.01)	5/10	0.56 (0.18–1.73)
Domestic services (95.0)	5/10	0.71 (0.23–2.12)	5/10	0.60 (0.19–1.83)
Domestic services (95.0Z)	5/10	0.70 (0.23–2.12)	5/10	0.59 (0.19–1.83)

Significant differences in bold.^1^For ISCO-1968 codes, in total: 428/652 for one and two digits, 426/648 for three digits, and 402/626 for four and five digits in crude analyses.

^2^For ISCO-1968 codes, in total: 425/645 for one and two digits, 423/641 for three digits, and 400/619 for four and five digits in adjusted analyses.

^3^For NAF-1999 codes, in total: 425/640 for one and two digits, 394/597 for three digits, and 357/533 for four digits in crude analyses.

^4^For NAF-1999, in total: 422/633 for one and two digits, 393/591 for three digits, and 356/527 for four digits in adjusted analyses.

^5^Adjusted for sibship size, born from multiple pregnancies, personal history of testicular trauma, family history of testicular cancer, and family history of cryptorchidism.

**Table 5:** Odds ratios (OR) and 95% confidence intervals (CI) for TGCT associated with mother’s job (ISCO-1968) and industry sector (NAF-1999) at birth, according to TGCT histological subtypes, case–control study, *N* = 1,124, France, 2015–2018.

		Non-seminomas		Seminomas			P-HET**
	*N* cases/ *N* controls	Crude OR (95% CI)	Adjusted OR* (95% CI)	*N* cases/ *N* controls	Crude OR (95% CI)	Adjusted OR* (95% CI)	
ISCO-1968 codes							
**Professional, technical and related workers (0/1)**	42/137	1.37 (0.89–2.09)	1.36 (0.88–2.10)	41/137	0.97 (0.65–1.45)	0.99 (0.66–1.50)	0.30
Medical, dental, veterinary, and related workers (0–6/0–7)	9/35	1.07 (0.48–2.39)	1.14 (0.51–2.55)	6/35	0.64 (0.28–1.49)	0.58 (0.23–1.43)	0.27
Teachers (1–3)	18/41	**1.93 (1.02**–**3.64)**	1.91 (1.00–3.63)	13/41	0.96 (0.50–1.87)	1.06 (0.54–2.10)	0.22
Secondary education teachers (1–32)	11/14	**4.91 (1.98**–**12.19)**	**4.67 (1.87**–**11.67)**	7/14	1.40 (0.53–3.70)	1.47 (0.54–4.00)	0.09
Primary education teachers (1–33)	5/19	1.01 (0.35–2.90)	0.95 (0.32–2.80)	5/19	0.86 (0.31–2.35)	0.98 (0.35–2.72)	0.96
First-level education teacher (1–33.20)	5/19	1.04 (0.36–3.02)	0.97 (0.32–2.91)	5/19	0.89 (0.32–2.43)	1.02 (0.37–2.84)	0.94
**Clerical and related workers (3)**	35/123	1.04 (0.67–1.61)	0.93 (0.59–1.48)	48/123	1.20 (0.81–1.76)	1.14 (0.76–1.71)	0.52
Stenographers, typists, and card-and tape-punching machine operators (3–2)	8/45	0.77 (0.35–1.70)	0.63 (0.28–1.43)	17/45	1.08 (0.59–1.97)	1.01 (0.54–1.87)	0.37
Stenographers, typists, and teletypists (3–21)	8/45	0.77 (0.35–1.71)	0.64 (0.28–1.44)	17/45	1.07 (0.59–1.95)	1.00 (0.54–1.85)	0.39
Stenographer-typist (General) (3–21.10)	5/32	0.64 (0.24–1.71)	0.55 (0.20–1.51)	12/32	1.48 (0.77–2.82)	1.01 (0.49–2.10)	0.33
Clerical and related workers not elsewhere classified (3–9)	20/49	1.43 (0.81–2.54)	1.30 (0.71–2.38)	19/49	1.26 (0.71–2.21)	1.13 (0.62–2.04)	0.74
Correspondence and reporting clerks (3–93)	18/41	1.70 (0.92–3.15)	1.58 (0.84–2.97)	16/41	1.25 (0.68–2.31)	1.09 (0.57–2.07)	0.42
Office clerk (general) (3–93.10)	14/35	1.64 (0.82–3.26)	1.53 (0.76–3.11)	15/35	1.48 (0.77–2.82)	1.21 (0.61–2.39)	0.63
**Sales workers (4)**	9/35	0.93 (0.42–2.04)	0.98 (0.44–2.17)	11/35	1.04 (0.51–2.11)	1.01 (0.49–2.10)	0.96
**Service workers (5)**	19/73	0.76 (0.43–1.32)	0.78 (0.44–1.37)	22/73	0.94 (0.56–1.58)	0.96 (0.56–1.63)	0.60
**Production and related workers, transport equipment operators, and laborers (7/8/9)**	6/44	0.43 (0.17–1.06)	0.45 (0.18–1.12)	10/44	0.64 (0.31–1.32)	0.72 (0.34–1.52)	0.43
Miners, quarrymen, well drillers, and related workers (7–1)	7/24	1.30 (0.52–3.22)	1.45 (0.58–3.63)	5/24	0.85 (0.33–2.16)	0.74 (0.27–2.04)	0.33
NAF-1999 codes							
**Manufacturing (15–37)**	8/43	0.64 (0.28–1.44)	0.62 (0.27–1.43)	10/43	0.72 (0.35–1.48)	0.77 (0.37–1.62)	0.69
**Wholesale and retail trade, repair of motor vehicles, motorcycles**,**and personal and household goods (50, 51, 52)**	8/43	0.64 (0.28–1.42)	0.72 (0.32–1.63)	14/43	0.95 (0.50–1.79)	1.00 (0.52–1.93)	0.54
Retail and repair of household goods (52)	7/39	0.60 (0.26–1.41)	0.70 (0.29–1.66)	12/39	0.87 (0.44–1.72)	0.98 (0.49–1.96)	0.54
**Real estate, renting, and business activities (70, 71, 72, 73, 74)**	9/18	1.47 (0.61–3.54)	1.39 (0.55–3.52)	6/18	1.13 (0.44–2.93)	1.14 (0.44–3.00)	0.78
**Education (80)**	19/47	1.64 (0.89–3.03)	1.62 (0.87–3.00)	16/47	1.04 (0.57–1.90)	1.14 (0.62–2.13)	0.43
Primary education (80.1)	7/22	1.14 (0.46–2.86)	1.08 (0.42–2.76)	7/22	0.96 (0.4–2.310)	1.09 (0.44–2.67)	0.99
Primary education (80.1Z)	7/22	1.14 (0.45–2.89)	1.08 (0.42–2.80)	7/22	0.86 (0.35–2.10)	0.99 (0.40–2.44)	0.89
Secondary education (80.2)	10/14	**3.86 (1.51**–**9.85)**	**3.50 (1.36**–**9.01)**	8/14	1.82 (0.71–4.64)	1.88 (0.72–4.93)	0.37
**Health and social work (85)**	19/91	0.77 (0.45–1.33)	0.76 (0.44–1.34)	28/91	1.09 (0.69–1.74)	1.00 (0.62–1.61)	0.48
Activities for human health (85.1)	10/62	0.62 (0.30–1.27)	0.66 (0.32–1.36)	16/62	0.84 (0.47–1.52)	0.82 (0.45–1.51)	0.64
Hospital activities (85.1A)	7/36	0.63 (0.26–1.54)	0.67 (0.27–1.63)	12/36	1.16 (0.58–2.35)	1.10 (0.53–2.28)	0.39
Social work activities (85.3)	5/16	0.88 (0.30–2.58)	0.93 (0.32–2.72)	5/16	1.22 (0.43–3.46)	1.12 (0.38–3.32)	0.81

Significant differences in bold.*Adjusted for sibship size, born from multiple pregnancies, personal history of testicular trauma, family history of testicular cancer, and family history of cryptorchidism.

**p-value for heterogeneity derived from the likelihood ratio test, comparing seminoma versus non-seminoma tumours in adjusted models.

A vast majority of analyzed occupations did not show any association between mother’s industry at birth (NAF-1999) and TGCT and subtypes, with the exception of statistically significant increases in overall and non-seminoma TGCT risks for “Secondary education (80.2)” (OR = 2.35, CI 1.13–4.88 and 3.50, CI 1.36–9.01, respectively) (Tables 4, 5).

### 3.4 Sensitivity analysis

In the sensitivity analysis, excluding TGCT cases with a personal history of cryptorchidism and unconfirmed cases, the positive associations previously observed remained for paternal and maternal employments and industries at birth. An inverse association was observed for paternal “Professional, technical, and related workers (0/1)” after exclusion of TGCT cases with a personal history of cryptorchidism (Supplementary Table S3). A significant association was observed for maternal “Auditor (1–10.20)” (OR = 2.51, CI 1.11–5.70), after excluding TGCT cases not confirmed by pathology report (Supplementary Table S4).

From occupational information reported by mothers, a statistically significant increase was seen in sensitivity analysis for paternal industries “Crops (01.1)” (OR = 3.29, CI 1.04–10.44). The other associations observed from the data reported by participants were not confirmed in this sensitivity analysis (Supplementary Table S5). No significant association was observed for mother’s employment and industries at birth (Supplementary Table S6).

After adjustment for age as a continuous variable, positive associations were confirmed for the paternal jobs of “Protective service workers (5–8)” (OR = 2.05, CI 1.01–4.16), “Transport equipment operators (9–8)” 2.06 (1.22–3.50) and “Specialized farmers (6–12)” (OR = 2.62, CI 1.01–6.82). Moreover, a positive association was found for “Motor vehicle drivers (9–85)” (OR = 1.78, CI 1.03–3.09), and an inverse association for “Managers (2–1)” (OR = 0.50, 0.27–0.95) (Supplementary Table S7). The same associations as in the main analyses were observed for mothers’ jobs and industries at birth (Supplementary Table S8).

## 4 Discussion

This study is one of the rare studies, and the first study in France, investigating parental job at birth in patients with TGCT, also considering histological subtypes.

### 4.1 Key findings

In this study, the results suggest that certain parental jobs or industries in perinatal life may be involved in the TGCT development of adult sons. Some positive associations were observed for paternal (service worker, protective service worker, transport equipment operator) and maternal employment (secondary education teacher, secondary education). The risk of seminomas was increased for the above-mentioned paternal jobs and for working as specialized farmer (a category including field crop farmers, vineyard farmers, dairy farmers, and horticultural farmers, for example), but few significant associations were found for any industry in general or for any jobs among mothers. Non-seminomas were associated with working in public administration and defence, compulsory social security for fathers, and working as a secondary education teacher or in secondary education for mothers. These differences between histological subtypes were compatible with random fluctuations. An inverse association has been observed for paternal jobs at birth, such as material handling and related equipment operators, dockers, and freight handlers. When comparing the results of the analyses between jobs and industries, many of the associations, positive or inverse, did not intersect for fathers. Nevertheless, the positive associations or trends between the risk of TGCT or seminoma and working as a farmer, a specialized farmer, or, as observed only in sensitivity analyses from mothers’ data, in agriculture, hunting, and related service activity, in crops, cereals, and industrial crops may suggest a possible link between agricultural work, related to potential occupational exposure to pesticides, for example, and the risk of developing TGCT. For jobs and industries among mothers, the positive associations are with secondary education.

### 4.2 Literature data

Only a few studies have investigated pre- and perinatal parental employment and the risk of developing TGCT in adulthood, and robust associations are scarce and sometimes contradictory (42). One case–control study on mothers and fathers’ employment around pregnancy, including 343 cases and 524 controls, found positive associations with fathers working as wood processors (OR = 10.46, CI 1.20–91.14), metalworkers (OR = 3.28, CI 1.03–10.52), stationary engineers (OR = 1.05, CI 1.05–11.87), employees of the food products (OR = 2.79, CI 1.34–5.79), metal products (OR = 5.77, CI 1.53–21.77), and food and beverage service (OR = 4.36, CI 1.50–12.63) industries. No association was seen regarding mothers’ occupations (41). In the NORD-TEST study (58), a registry-based case–control study in Sweden, Finland, and Norway, including 8,112 TGCT cases and 26,264 matched controls, a decreased risk of TGCT in offspring (OR = 0.85, CI 0.75–0.96) was observed for paternal wood-related jobs.

Numerous studies suggest that seminomatous and non-seminomatous tumors derive from a common precursor (GCNIS), which may be part of testicular dysgenesis syndrome. This supports the theory of an *in utero* onset of the disease, for which no genetic mutation has been clearly identified, that may be related to possible pre- or perinatal exposure, such as EDC (10, 59, 60). Arguments have been put forward that non-seminomatous tumors are more likely to be involved in early exposure due to their earlier age of onset (14). However, in the present study and in previous studies having investigated seminomas and non-seminomas separately, no robust specific risk factors have been found, and it is suggested that both histological subtypes share important aetiological factors (42, 61).

### 4.3 Hypothesis on occupational exposures

#### 4.3.1 Paternal occupational exposures

Working as a service worker (involving use of specialized products or maintenance and cleaning tasks in cooks, waiters, hairdressers, and beauticians), protective service worker (firefighters, factory guards), transport equipment operators, or in the publishing, printing, and reproduction industries are jobs and activity sectors involving, among others, exposure to solvents (62). Some of these parental occupations at birth have previously been suspected to be related to the development of TGCT in children and adults (42), as well as prenatal exposure to solvents (63, 64), particularly seminomas (44). Among sons, an increased risk of developing TGCT was observed in the TESTIS population in association with some occupations that could be related to solvent exposures (subjects employed in the trade, motor vehicle repair, and household goods industries), which may support the hypothesis of a link between TGCT and occupational exposure to solvents (65). However, some studies do not show the risk increases described in our study, for example among protective service workers (66).

The increased risk of developing TGCT in adulthood observed when the father worked as a protective service worker (a job category including firefighters, waiters, and cooks, for example) or as a transport equipment operator (a job category including locomotive driver, motor vehicle driver, etc.) could raise questions about the impact of exposure to polycyclic aromatic hydrocarbons or combustion products, which are known carcinogens at multiple sites and EDC (39, 67). However, to the best of our knowledge, the link between this prenatal exposure and the risk of TGCT has not been studied so far. Previous studies suggest a link between working as a firefighter and the development of TGCT, but without specifying which of the numerous occupational exposures in this job category would be involved (68–72).

Early exposure to heavy metals has been suggested to increase the risk of TGCT in previous publications (41, 44, 64, 73). Working as protective service workers, transport equipment operators, specialized farmers in publishing, printing, reproduction, or crop professions or industries may lead to exposure to lead (fuel, bullets, printing, etc.), copper (organic pesticide), or cadmium (printing) in the parents.

Pesticide exposure is one of the most frequently investigated prenatal exposures in terms of the risk of developing TGCT in adulthood, but the results of studies are divergent (15, 45, 74). Here, the risk of developing TGCT in adulthood was increased when the father had a job as specialized farmer (particularly seminomas) or worked in crops (all histological subtypes in sensitivity analysis from data reported by mothers), suggesting a possible link between perinatal pesticide exposure and TGCT risk, while our results across farming-related jobs seemed inconsistent. Among sons, an increased risk of developing TGCT was observed in the TESTIS population in association with having personally worked as a farmer, which appears to be consistent with our results (65). Moreover, many pesticides, such as organochlorines, have endocrine disruptive properties (75), which have been suggested to be implicated in the development of testicular dysgenesis syndrome and TGCT (15, 75).

#### 4.3.2 Maternal occupational exposures

Maternal occupational exposures in secondary education that may favor the development of overall TGCT and non-seminomas in adulthood do not appear to be evident. To the best of our knowledge, a link between mother’s teaching profession and the risk of TGCT in adulthood has not been observed in previous studies. However, a periodicity concerning the month of birth has been observed (57), with more men born in May, August, and January showing TGCT in adulthood. The sensitive period for the development of GCNIS *in utero* is the embryonic stage, i.e., the first trimester of pregnancy (32). For births in May, August, and January, this would correspond to critical periods of exposure during pregnancy from August to January and from April to June. The hypothesis underlying this observation, according to the authors, would be a rhythmicity linked to school periods or seasonal infectious epidemics (57). Two meta-analyses found an association between TGCT and Epstein–Barr virus (OR = 7.38, CI 1.89–28.75, *p* = 0.004 (76); OR = 4.80, CI 0.98–23.54 (77)) and divergent results for cytomegalovirus and parvovirus B19. Of note, these studies do not concern infections during pregnancy. Potential maternal occupational exposure to chemicals and semi-organic volatile compounds may occur in schools (through use for science or art classrooms, use in building materials, school equipment, cleaning, and pest control), including phthalates (78).

### 4.4 Strengths and limitations

Parental employment data were collected from mothers to minimize recall bias and facilitate the collection of employment data for the biological fathers. Yet, the two-stage recruitment process of case/control mothers/relatives in the present study resulted overall in a moderate participation rate of mothers/relatives (50% of the total sample), leading to a lack of power and concerns of potential recall bias when limiting the analyses to cases and controls with participating mothers. To limit recall and social desirability bias, cases and controls, as well as the subjects’ mothers, were given a document to prepare for the telephone interview regarding their professional and residential history and were invited to bring their health records to the interview. Yet, we cannot exclude that social desirability bias may apply to responses to items such as fertility, medical history, smoking, alcohol consumption, and substance use, which constitutes a limitation in the present study (79–82). Pre- and perinatal factors of participants and the socio-economic status of mothers at birth were similar between the groups of subjects with interviewed and non-interviewed mothers (48). We used the subsample of participants with interviewed mothers to study the agreement between the occupational data collected from the sons and from their mothers. Because of the good agreement, it appeared justified to take into account the sons’ data and thus double the number of participants, increasing the power of our study (83). The analysis of parental jobs at birth reported by the mothers showed only one association consistent with the results on the ISCO-68 and NAF-1999 codes of the sons’ data. Categories that appeared to be associated with the main analyses or from the mothers’ data could not be confirmed in the other analysis because of an insufficient number of subjects, whether the data came from participants or mothers. However, the positive association observed after adjustment from the mothers’ data for paternal jobs or industries “Crops” seems to be—focusing on the field of activity—in accordance with “Specialised farmers” found significant from the sons’ data.

To observe the relationship between parental jobs at birth and TGCT in offspring, the use of two nomenclatures based on job and industry categories and subcategories appeared complementary but involved multiple analyses. This limitation has been previously discussed (65). Although our results may be interpreted with caution, overall they appear to be consistent with the literature.

Endocrine disruptors are likely to be involved in the development of TGCTs (15, 20, 22). Due to their toxicological characteristics, including low-dose effects, non-monotonic dose-effect relationships, and sensitivity to the cocktail effect (22, 23), we cannot exclude that other environmental, domestic, or even occupational exposures, whether these exposures are concomitant or not, depending on half-lives or delayed effects, may participate in the development of TGCT in the offspring through additive or synergistic relationship with the occupational exposures appearing to be associated with TGCT here (15, 22, 23). However, a recent meta-analysis studying the link between exposure to EDC and the risk of TGCT highlighted that maternal, but not postnatal adult male, EDC exposures were consistently associated with a higher risk of TGCT, particularly of non-seminomas (22), which confirms the relevance of our study and our results. Furthermore, the assessment of cocktail effects and their involvement in health issues remains a methodological challenge (84, 85), which is reflected in the fact that this meta-analysis was unable to identify any study looking at mixtures of substances (22). Identifying jobs possibly involved in TGCT development could be an opportunity to assess the link between global human exposures in real situations, rather than individual compounds. Birth was chosen as the period of parental occupational exposure of interest, being a proxy of periconceptional, gestational, and first 1,000 days of life exposures, key periods in child development, assuming, on the basis of employment data at the time of the birth, that the parents remain in the same job or field of activity overall (86). This could be a preliminary work to identify exposures at risk in the early childhood period, which could be completed later by studies on each of these periods independently.

## 5 Conclusion

Our study suggests that parental jobs or industries in perinatal life, such as paternal jobs at birth as a service worker, protective service worker, transport equipment operator, specialized farmer, and maternal jobs as secondary education teachers or working in secondary education at birth, may be related to the development of adult TGCT in their sons, which appears consistent with some previous studies, with specific features depending on the histological type.

Further studies on the different occupational exposures related to these jobs and industries of the parents at birth are needed, as we have done or plan to do with solvents, pesticides, or heavy metals (87). In addition, the combined effect of early life exposure, leading to the development of GCNIS, and later life exposure in adolescence or adulthood, acting as a trigger for the evolution of GCNIS to TGCT, should be taken into account in future studies.

## Data availability statement

The datasets presented in this article are not readily available because data are available on reasonable request. The data collection process is described in the method section of this article. Requests to access the datasets should be directed to BF beatrice.fervers@lyon.unicancer.fr.

## Ethics statement

The studies involving humans were approved by the French Ethics Committee (ref. no. A14-94), the French National Agency for the Safety of Medicines and Health Products (ref. no. 140184B-12), and the IARC Ethics Committee (ref. no. 14–26) and declared to the Commission nationale de l’Informatique et des Libertés (MR-001, ref. no. 2016–177). The studies were conducted in accordance with the local legislation and institutional requirements. The participants provided their written informed consent to participate in this study.

## Author contributions

AP: Conceptualization, Formal analysis, Funding acquisition, Methodology, Writing – original draft, Writing – review & editing. AD: Conceptualization, Data curation, Formal analysis, Funding acquisition, Investigation, Methodology, Writing – review & editing. FD: Data curation, Software, Writing – review & editing. MG: Validation, Writing – review & editing. AC: Project administration, Validation, Writing – review & editing. ML: Data curation, Formal analysis, Software, Writing – review & editing. BD: Data curation, Writing – review & editing. HK: Investigation, Methodology, Resources, Validation, Writing – review & editing. JHS: Methodology, Supervision, Writing – review & editing. RB: Data curation, Validation, Writing – review & editing. OP: Funding acquisition, Project administration, Writing – review & editing. HB: Data curation, Resources, Writing – review & editing. CH: Resources, Writing – review & editing. VL-C: Resources, Writing – review & editing. SV: Resources, Writing – review & editing. LB: Resources, Writing – review & editing. AO: Conceptualization, Methodology, Supervision, Validation, Writing – review & editing. JAS: Conceptualization, Methodology, Supervision, Validation, Writing – review & editing. BF: Conceptualization, Funding acquisition, Investigation, Methodology, Project administration, Supervision, Validation, Writing – review & editing. BC: Conceptualization, Investigation, Methodology, Supervision, Validation, Writing – review & editing.

## Collaborators

TESTIS Study Group: Aude Fléchon and Elodie Belladame (Centre Léon Bérard, Lyon). Véronique Drouineaud, Céline Chalas, Aurélie Vincent, Anne-Sophie Gille, Laurianne Kremer, Myriam Virlouvet, Lucile Ferreux, Guillemette Perier, Pauline Peretout, and Diane Rivet (Cochin Hospital, Paris); Rachel Levy and Isabelle Berthaut (Tenon Hospital, Paris); Florence Eustache, Nathalie Sermonande, Marine Durand, and Charlène Harbemont (Jean Verdier Hospital, Paris); Jeanne Perrin, Jacqueline Saias-Magnan, Carole Daoud-Deveze, and Catherine Metzler-Guillemain (La Conception Hospital, Marseille); Myriam Daudin and François Isus (Paule de Viguier Hospital,Toulouse); Florence Brugnon, Valérie Bruhat, and Cyril Bouche (Estaing Hospital, Clermont-Ferrand); Isabelle Koscinski, Cécile Greze, Françoise Schmitt, Marius Teletin, and Nathalie Guyomard (Obstetric Medico-Surgical Centre, Strasbourg); Aline Papaxanthos, Volcy Soula, and Lucie Chansel (Pellegrin maternity Hospital, Bordeaux); Sandrine Giscard d’Estaing, Pascale Dehee, and Delphine Yalcinkaya (Femme-Mère-Enfant Hospital, Lyon); Céline Bouillon, Cynthia Frapsauce, Anne Viallon, and Catherine Guerin (Bretonneau Hospital, Tour); Berengere Ducrocq and Florian Dossou Gbete (Calmette Hospital, Lille); Marie-Ange Clarotti, Amélie Ancelle, and Emeline Bovet-Courtois (Caen Hospital, Caen); Oxana Blagosklonov and Séverine Bey (Jean Minjoz Hospital, Besançon); Célia Ravel, Guilhem Jouve, Laurent Vandenbroucke, and Agnès Letremy (South Hospital, Rennes); Patricia Fauque, Julie Barberet, and Céline Bruno (Dijon Hospital, Dijon); Stéphanie Lattes Emmanuelle Thibault, Pierre Besnier, and Adrien Perche (l’Archet Hospital, Nice); Catherine Diligent (Regional University Maternity Hospital, Nancy); Anna Gala and Christelle Saintpeyre (Arnaud de Villeneuve Hospital, Montpellier); Sylvianne Hennebicq and Julien Bessonnat (Couple-Enfant Hospital, Grenoble); Marie-Claude Blocquaux (Maison Blanche Hospital, Reims).
