# Supplementary material for: Parental occupations at birth and risk of adult testicular germ cell tumors in offspring: a French nationwide case–control study
Source: Front Public Health. 2024 Jan 16;11:1303998. doi: 10.3389/fpubh.2023.1303998 (PMC10825020; doi:10.3389/fpubh.2023.1303998)
Supplement: Supplementary file 1 [file Data_Sheet_1.pdf]

Supplementary Material

Table S1. Agreement between parental employments at birth collected from the participants and the ones collected from the mothers, case-control study, France, 2015-2018.

	N	(%)	Kappa
**Paternal jobs at birth**			
Concordant ISCO-1968 codes (N=384)			
1 digit	294	(75.6)	0.72
2 digits	263	(67.6)	0.67
3 digits	234	(60.2)	0.60
4 digits	197	(50.6)	0,50
5 digits	197	(50.6)	0.50
Concordant NAF-1999 codes (N=298)			
2 digits	237	(76.2)	0.75
3 digits	214	(68.8)	0.68
4 digits	191	(61.4)	0.61
**Maternal jobs at birth**			
Concordant ISCO-1968 codes (N=276)			
1 digit	231	(80.8)	0.76
2 digits	190	(66.4)	0,64
3 digits	177	(61.9)	0.60
4 digits	163	(57.0)	0.55
5 digits	163	(57.0)	0.55
Concordant NAF-1999 codes (N=159)			
2 digits	145	(87.3)	0.85
3 digits	136	(81.9)	0.80
4 digits	130	(78.3)	0.77
