# Supplementary material for: Parental occupations at birth and risk of adult testicular germ cell tumors in offspring: a French nationwide case–control study
Source: Front Public Health. 2024 Jan 16;11:1303998. doi: 10.3389/fpubh.2023.1303998 (PMC10825020; doi:10.3389/fpubh.2023.1303998)
Supplement: Supplementary file 2 [file Data_Sheet_2.pdf]

Supplementary material.

Table S2. Parental employment at birth (ISCO-68 category), collected from sons, according to mother's participation status in the TESTIS study.

ISCO-68 category	Interviewed mothers (N=547)		Non interviewed mothers (N=577)		p (Khi-2)
	N	%	N	%	0,01
Paternal jobs at birth					
Professional, Technical and Related Workers (0)	103	19%	69	12%	
Professional, Technical and Related Workers (1)	56	10%	38	7%	
Administration and managerial workers (2)	33	6%	29	5%	
Clerical and related workers (3)	42	8%	48	8%	
Sales workers (4)	29	5%	48	8%	
Service workers (5)	31	6%	40	7%	
Agricultural, animal husbandry and forestry workers, fishermen and hunters (6)	36	7%	33	6%	
Production and related workers, transport equipment operators and labourers (7)	20	4%	34	6%	
Production and related workers, transport equipment operators and labourers (8)	67	12%	91	16%	
Production and related workers, transport equipment operators and labourers (9)	87	16%	99	17%	
Unemployed	18	3%	15	3%	
Missing data	22	4%	25	4%	
Students	0	0%	2	0%	
Military personnel	3	1%	6	1%	
N Total	547		577		
Maternal jobs at birth					
Professional, Technical and Related Workers (0)	59	11%	35	6%	
Professional, Technical and Related Workers (1)	73	13%	58	10%	
Administration and managerial workers (2)	12	2%	5	1%	
Clerical and related workers (3)	113	21%	109	19%	
Sales workers (4)	30	5%	29	5%	
Service workers (5)	58	11%	64	11%	
Agricultural, animal husbandry and forestry workers, fishermen and hunters (6)	13	2%	11	2%	
Production and related workers, transport equipment operators and labourers (7)	11	2%	16	3%	<0,0001
Production and related workers, transport equipment operators and labourers (8)	9	2%	7	1%	
Production and related workers, transport equipment operators and labourers (9)	7	1%	15	3%	
Unemployed	137	25%	215	37%	
Missing data	25	5%	11	2%	
Students	0	0%	2	0%	
Military personnel	0	0%	0	0%	
N Total	547		577		
