# Supplementary material for: Parental occupations at birth and risk of adult testicular germ cell tumors in offspring: a French nationwide case–control study
Source: Front Public Health. 2024 Jan 16;11:1303998. doi: 10.3389/fpubh.2023.1303998 (PMC10825020; doi:10.3389/fpubh.2023.1303998)
Supplement: Supplementary file 3 [file Data_Sheet_3.pdf]

Supplementary Material

Table S3. Odds ratios (OR) and 95% confidence intervals (CI) for TGCT associated with father's job (ISCO-1968) and industry sector (NAF-1999) at birth, overall, sensitivity analyses, case-control study, N=1124, France, 2015-2018.

	**Excluding TGCT cases with personal history of cryptorchidism (N=1084)**				**Excluding TGCT cases not confirmed by pathology report (N=1081)**			
	**N cases / N controls**	**Crude OR (95% CI)**	**N cases / N controls**	**Adjusted OR* (95% CI)**	**N cases / N controls**	**Crude OR (95% CI)**	**N cases / N controls**	**Adjusted OR* (95% CI)**
**ISCO-1968 CODES**								
**Professional, Technical and Related Workers (0/1)**	76 / 175	0.74 (0.54-1.01)	75 / 173	**0.69 (0.50-0.96)**	82 / 175	0.81 (0.59-1.10)	81 / 173	0.76 (0.56-1.05)
Architects, engineers and related technicians (0-2/0-3)	28 / 58	0.87 (0.54-1.42)	28 / 57	0.83 (0.51-1.37)	29 / 58	0.96 (0.59-1.54)	29 / 57	0.92 (0.56-1.51)
Draughtsmen (0-32)	6 / 14	0.62 (0.23-1.67)	6 / 14	0.63 (0.22-1.76)	7 / 14	0.74 (0.29-1.90)	7 / 14	0.78 (0.30-2.06)
Electrical and electronics engineering (0-34)	5 / 7	1.28 (0.39-4.20)	5 / 7	0.95 (0.28-3.28)	5 / 7	1.35 (0.41-4.39)	5 / 7	0.95 (0.28-3.28)
Medical, dental, veterinary and related workers (0-6/0-7)	8 / 33	0.40 (0.18-0.87)	8 / 33	0.42 (0.19-0.94)	11 / 33	0.53 (0.26-1.07)	11 / 33	0.56 (0.27-1.14)
Medical doctors (0-61)	7 / 20	0.56 (0.23-1.35)	7 / 20	0.59 (0.24-1.44)	10 / 20	0.77 (0.35-1.69)	10 / 20	0.80 (0.36-1.77)
Specialized physician (0-61.20)	5 / 8	0.94 (0.28-3.10)	5 / 8	1.12 (0.33-3.78)	5 / 8	1.03 (0.31-3.40)	5 / 8	1.24 (0.37-4.15)
Statisticians and related technicians (0-8)	8 / 12	0.96 (0.37-2.47)	8 / 12	0.92 (0.35-2.42)	10 / 12	1.19 (0.49-2.88)	10 / 12	1.18 (0.48-2.90)
Systems analysts (0-83)	7 / 8	1.08 (0.36-3.22)	7 / 8	1.11 (0.36-3.42)	8 / 8	1.28 (0.45-3.65)	8 / 8	1.33 (0.45-3.92)
Systems analyst (0-83.10)	7 / 8	1.18 (0.40-3.50)	7 / 8	1.20 (0.39-3.72)	8 / 8	1.46 (0.51-4.16)	8 / 8	1.53 (0.52-4.55)
Accountants (1-1)	7 / 12	1.20 (0.46-3.11)	7 / 11	1.42 (0.53-3.78)	5 / 12	0.78 (0.27-2.25)	5 / 11	0.93 (0.31-2.76)
Accountants (1-10)	7 / 12	1.15 (0.44-2.99)	7 / 11	1.36 (0.51-3.60)	5 / 12	0.74 (0.26-2.15)	5 / 11	0.88 (0.30-2.61)
Teachers (1-3)	11 / 29	0.75 (0.37-1.54)	10 / 29	0.64 (0.30-1.38)	14 / 29	0.91 (0.47-1.76)	13 / 29	0.74 (0.36-1.52)
Secondary education teachers (1-32)	7 / 11	1.17 (0.44-3.09)	7 / 11	1.02 (0.37-2.82)	8 / 11	1.23 (0.48-3.13)	8 / 11	1.03 (0.38-2.81)
**Administrative and managerial workers (2)**	21 / 41	0.80 (0.46-1.40)	21 / 41	0.80 (0.45-1.41)	18 / 41	0.70 (0.39-1.26)	18 / 41	0.68 (0.37-1.23)
Managers (2-1)	18 / 41	0.67 (0.37-1.20)	18 / 41	0.66 (0.36-1.20)	15 / 41	0.57 (0.31-1.06)	15 / 41	0.54 (0.28-1.02)
General managers (2-11)	5 / 8	1.11 (0.36-3.48)	5 / 8	1.10 (0.33-3.61)	-	-	-	-
General manager (2-11.10)	5 / 8	1.13 (0.36-3.53)	5 / 8	1.16 (0.35-3.82)	-	-	-	-
Managers not elsewhere classified (2-19)	10 / 23	0.64 (0.30-1.39)	10 / 23	0.65 (0.30-1.44)	9 / 23	0.60 (0.27-1.33)	9 / 23	0.59 (0.26-1.34)
Other managers (2-19.90)	5 / 15	0.48 (0.17-1.36)	5 / 15	0.43 (0.15-1.26)	-	-	-	-

**Clerical and related workers (3)**	33 / 53	1.11 (0.69-1.77)	33 / 52	1.13 (0.70-1.83)	30 / 52	0.99 (0.61-1.60)	30 / 51	1.03 (0.63-1.69)
Bookkeepers, cashiers and related workers (3-3)	11 / 12	1.68 (0.72-3.96)	11 / 11	1.58 (0.64-3.88)	10 / 11	1.54 (0.64-3.73)	10 / 10	1.41 (0.56-3.58)
Bookkeepers and cashiers (3-31)	5 / 5	1.78 (0.50-6.28)	5 / 5	1.57 (0.44-5.63)	5 / 5	1.82 (0.51-6.47)	5 / 5	1.61 (0.44-5.84)
Bookkeepers, cashiers and related workers not elsewhere classified (3-39)	6 / 7	1.58 (0.50-5.00)	6 / 6	1.57 (0.45-5.47)	5 / 6	1.28 (0.38-4.35)	5 / 5	1.21 (0.32-4.54)
Finance clerk (3-39.40)	6 / 7	1.50 (0.47-4.77)	6 / 6	1.40 (0.41-4.87)	5 / 6	1.26 (0.37-4.28)	5 / 5	1.14 (0.31-4.28)
Mail distribution clerks (3-7)	5 / 5	2.03 (0.58-7.07)	5 / 5	2.12 (0.57-7.82)	-	-	-	-
Mail distribution clerks (3-70)	5 / 5	1.99 (0.57-6.94)	5 / 5	2.06 (0.56-7.57)	-	-	-	-
Clerical and related workers not elsewhere classified (3-9)	11 / 27	0.68 (0.33-1.41)	11 / 27	0.74 (0.36-1.53)	11 / 27	0.70 (0.34-1.44)	11 / 27	0.75 (0.36-1.55)
Stock clerks (3-91)	-	-	-	-	-	-	-	-
Correspondence and reporting clerks (3-93)	6 / 11	0.98 (0.36-2.72)	6 / 11	1.00 (0.36-2.79)	6 / 11	1.01 (0.37-2.79)	6 / 11	0.99 (0.35-2.79)
Office clerk (general) (3-93.10)	6 / 10	1.10 (0.39-3.12)	6 / 10	1.10 (0.38-3.15)	6 / 10	1.13 (0.40-3.19)	6 / 10	1.06 (0.37-3.07)
**Sales Workers (4)**	31 / 42	1.21 (0.73-1.99)	31 / 40	1.37 (0.82-2.29)	30 / 42	1.25 (0.75-2.06)	30 / 40	1.41 (0.84-2.36)
Working proprietors (wholesale and retail trade) (4-1)	5 / 8	1.14 (0.36-3.58)	5 / 8	1.27 (0.39-4.08)	7 / 8	1.65 (0.58-4.70)	7 / 8	1.86 (0.64-5.41)
Working proprietors (wholesale and retail trade) (4-10)	5 / 7	1.24 (0.38-4.03)	5 / 7	1.37 (0.41-4.56)	7 / 7	1.81 (0.61-5.34)	7 / 7	2.01 (0.67-6.05)
Working proprietor (retail trade) (4-10.30)	5 / 6	1.31 (0.37-4.62)	5 / 6	1.45 (0.41-5.15)	7 / 6	1.94 (0.62-6.10)	7 / 6	2.12 (0.66-6.75)
Technical salesmen, commercial travellers and manufacturers' agents (4-3)	15 / 19	1.24 (0.60-2.57)	15 / 18	1.45 (0.69-3.06)	11 / 19	1.02 (0.47-2.23)	11 / 18	1.18 (0.53-2.63)
Technical salesmen and service advisers (4-31)	13 / 16	1.22 (0.56-2.65)	13 / 15	1.43 (0.64-3.17)	10 / 16	1.04 (0.46-2.36)	10 / 15	1.21 (0.52-2.82)
Technical salesman (4-31.20)	13 / 16	1.17 (0.53-2.56)	13 / 15	1.39 (0.61-3.13)	10 / 16	1.01 (0.45-2.29)	10 / 15	1.18 (0.50-2.77)
Salesmen, shop assistants and related workers (4-5)	6 / 8	1.12 (0.37-3.35)	6 / 8	1.14 (0.37-3.47)	6 / 8	1.12 (0.37-3.35)	6 / 8	1.16 (0.38-3.57)
Salesmen, shop assistants and demonstrators (4-51)	-	-	-	-	6 / 4	2.15 (0.59-7.87)	6 / 4	2.10 (0.56-7.89)
**Service Workers (5)**	33 / 34	**2.00 (1.19-3.36)**	34 / 33	**2.03 (1.20-3.45)**	34 / 33	**1.92 (1.15-3.21)**	34 / 33	**1.89 (1.12-3.20)**
Cooks, waiters, bartenders and related workers (5-3)	7 / 8	1.49 (0.53-4.22)	7 / 8	1.35 (0.47-3.90)	7 / 8	1.42 (0.50-4.00)	7 / 8	1.31 (0.45-3.80)
Protective service workers (5-8)	20 / 15	**2.45 (1.22-4.92)**	20 / 15	**2.43 (1.20-4.92)**	19 / 15	**2.31 (1.14-4.66)**	19 / 15	**2.26 (1.11-4.62)**
Policeman (5-82.20)	-	-	-	-	-	-	-	-
Protective service workers not elsewhere classified (5-89)	10 / 9	1.86 (0.73-4.75)	10 / 9	1.71 (0.67-4.39)	11 / 9	2.09 (0.84-5.21)	11 / 9	1.92 (0.76-4.83)
Other protective service workers (5-89.90)	8 / 9	1.53 (0.57-4.11)	8 / 9	1.33 (0.49-3.62)	8 / 9	1.56 (0.58-4.21)	8 / 9	1.40 (0.51-3.84)
**Agricultural, animal husbandry and forestry workers, fishermen and hunters (6)**	29 / 37	1.28 (0.77-2.15)	28 / 37	1.21 (0.71-2.06)	30 / 37	1.28 (0.77-2.13)	29 / 37	1.23 (0.73-2.08)
Farmers (6-1)	21 / 21	1.75 (0.93-3.31)	20 / 21	1.71 (0.88-3.31)	22 / 21	1.76 (0.94-3.31)	21 / 21	1.75 (0.91-3.34)

General farmers (6-11)	11 / 12	1.35 (0.58-3.17)	10 / 12	1.16 (0.47-2.87)	11 / 12	1.23 (0.53-2.87)	10 / 12	1.10 (0.45-2.68)
General farmer (6-11.10)	11 / 11	1.43 (0.59-3.46)	10 / 11	1.26 (0.49-3.19)	11 / 11	1.26 (0.52-3.04)	10 / 11	1.14 (0.45-2.86)
Specialized farmers (6-12)	10 / 8	2.41 (0.93-6.29)	10 / 8	**2.68 (1.01-7.11)**	11 / 8	**2.73 (1.07-6.97)**	11 / 8	**2.95 (1.14-7.67)**
Agricultural and animal husbandry workers (6-2)	6 / 10	0.98 (0.34-2.83)	6 / 10	0.94 (0.32-2.76)	6 / 10	0.92 (0.32-2.63)	6 / 10	0.92 (0.32-2.67)
**Production and Related Workers, Transport Equipment operators and labourers (7/8/9)**	141 / 234	1.01 (0.77-1.33)	140 / 233	1.02 (0.77-1.35)	139 / 232	0.99 (0.75-1.31)	138 / 231	1.00 (0.76-1.34)
Production supervisors and general foremen (7-0)	7 / 12	1.04 (0.40-2.68)	7 / 12	1.16 (0.44-3.04)	7 / 12	0.97 (0.37-2.54)	7 / 12	1.08 (0.41-2.88)
Production supervisors and general foremen (7-00)	7 / 12	1.00 (0.39-2.59)	7 / 12	1.11 (0.42-2.89)	7 / 12	0.93 (0.36-2.44)	7 / 12	1.03 (0.39-2.72)
Food and beverage processers (7-7)	7 / 16	0.71 (0.28-1.78)	7 / 16	0.67 (0.27-1.72)	8 / 16	0.86 (0.36-2.08)	8 / 16	0.82 (0.34-2.01)
Blacksmiths, toolmakers and machine-tool operators (8-3)	13 / 13	1.52 (0.69-3.36)	13 / 13	1.42 (0.62-3.25)	13 / 13	1.63 (0.74-3.62)	13 / 13	1.56 (0.68-3.56)
Machinery fitters, machine assemblers and precision-instrument makers [except electrical] (8-4)	20 / 33	0.95 (0.53-1.69)	19 / 33	0.88 (0.48-1.59)	16 / 33	0.77 (0.41-1.44)	15 / 33	0.73 (0.39-1.40)
Motor-vehicle mechanics (8-43)	8 / 10	1.22 (0.47-3.20)	8 / 10	1.09 (0.42-2.88)	5 / 10	0.75 (0.25-2.27)	5 / 10	0.76 (0.25-2.34)
Automobile mechanic (8-43.20)	6 / 6	1.64 (0.52-5.22)	6 / 6	1.43 (0.45-4.57)	-	-	-	-
Machinery fitters, machine assemblers and precision-instrument makers [except electrical] not elsewhere classified (8-49)	7 / 15	0.71 (0.28-1.78)	7 / 15	0.75 (0.30-1.89)	7 / 15	0.67 (0.27-1.69)	7 / 15	0.70 (0.28-1.78)
Electrical fitters and related electrical and electronics workers (8-5)	8 / 21	0.62 (0.27-1.44)	8 / 21	0.52 (0.22-1.24)	7 / 21	0.53 (0.22-1.27)	7 / 21	0.47 (0.19-1.15)
Electrical wiremen (8-55)	-	-	-	-	-	-	-	-
Plumbers, welders, sheet-metal and structural metal preparers and erectors (8-7)	13 / 21	1.05 (0.51-2.16)	13 / 21	1.08 (0.52-2.25)	11 / 21	0.87 (0.41-1.84)	11 / 21	0.86 (0.40-1.85)
Welders and flame-cutters (8-72)	5 / 4	1.50 (0.39-5.82)	5 / 4	1.35 (0.35-5.29)	-	-	-	-
Bricklayers, carpenters and other construction workers (9-5)	17 / 37	0.74 (0.40-1.34)	17 / 37	0.81 (0.44-1.50)	17 / 36	0.73 (0.40-1.34)	17 / 36	0.81 (0.44-1.49)
Bricklayers, stonemasons and tile setters (9-51)	13 / 13	1.45 (0.66-3.19)	13 / 13	1.62 (0.72-3.64)	13 / 13	1.44 (0.65-3.17)	13 / 13	1.59 (0.71-3.57)
Material handling and related equipment operators, dockers and freight handlers (9-7)	-	-	5 / 19	0.46 (0.17-1.26)	5 / 19	0.36 (0.13-1.03)		
Transport equipment operators (9-8)	29 / 30	1.69 (0.99-2.91)	29 / 29	**1.91 (1.09-3.34)**	32 / 29	**1.89 (1.11-3.22)**	32 / 28	**2.09 (1.20-3.65)**
Motor-vehicle drivers (9-85)	25 / 28	1.47 (0.83-2.60)	25 / 27	1.60 (0.89-2.88)	28 / 27	1.63 (0.94-2.86)	28 / 26	1.74 (0.98-3.11)
Lorry and van driver (local transport). (9-85.50)	11 / 11	1.51 (0.62-3.68)	11 / 11	1.40 (0.56-3.47)	12 / 11	1.63 (0.69-3.86)	12 / 11	1.40 (0.57-3.46)
Lorry and van driver (long-distance transport) (9-85.60)	-	-	-	-	-	-	-	-
**NAF-1999 CODES**								
**Agriculture, hunting and forestry (01, 02)**	30 / 38	1.32 (0.79-2.19)	29 / 38	1.30 (0.77-2.19)	31 / 38	1.33 (0.80-2.20)	30 / 38	1.32 (0.79-2.22)

Agriculture, hunting and related service activities (01)	27 / 32	1.47 (0.85-2.52)	26 / 32	1.48 (0.85-2.58)	28 / 32	1.47 (0.86-2.52)	27 / 32	1.50 (0.87-2.60)
Growing of crops combined with farming of animals (mixed farming) (01.3)	11 / 12	1.31 (0.56-3.07)	10 / 12	1.14 (0.46-2.85)	11 / 12	1.17 (0.50-2.74)	10 / 12	1.05 (0.42-2.58)
Growing of crops combined with farming of animals (mixed farming) (01.3Z)	11 / 12	1.44 (0.60-3.43)	10 / 12	1.28 (0.51-3.21)	11 / 12	1.27 (0.53-3.02)	10 / 12	1.17 (0.47-2.90)
**Manufacturing (15 to 37)**	57 / 105	0.89 (0.62-1.27)	57 / 103	0.87 (0.60-1.26)	56 / 105	0.87 (0.61-1.25)	56 / 103	0.85 (0.59-1.24)
Food industry (15)	6 / 14	0.62 (0.23-1.66)	6 / 13	0.64 (0.23-1.77)	6 / 14	0.64 (0.24-1.71)	6 / 13	0.66 (0.24-1.84)
Publishing, printing, reproduction (22)	8 / 5	**3.34 (1.06-10.52)**	8 / 5	**3.70 (1.15-11.84)**	8 / 5	**3.56 (1.13-11.24)**	8 / 5	**4.07 (1.27-13.07)**
Metalworking (28)	16 / 23	1.09 (0.56-2.11)	16 / 23	1.08 (0.55-2.14)	15 / 23	1.05 (0.54-2.07)	15 / 23	1.02 (0.51-2.06)
Manufacture of other transport equipment (35)	5 / 6	1.45 (0.43-4.89)	5 / 6	1.27 (0.36-4.49)	-	-	-	-
**Electricity, gas and water supply (40, 41)**	-	-	-	-	5 / 5	1.59 (0.45-5.66)	5 / 5	1.77 (0.49-6.42)
**Construction (45)**	41 / 69	0.97 (0.63-1.49)	41 / 69	1.02 (0.66-1.58)	38 / 68	0.87 (0.56-1.34)	38 / 68	0.91 (0.58-1.42)
Construction of building or civil engineering works (45.2)	13 / 22	0.95 (0.46-1.93)	13 / 22	1.15 (0.55-2.40)	13 / 22	0.91 (0.45-1.87)	13 / 22	1.05 (0.51-2.19)
Installation works (45.3)	9 / 17	0.83 (0.35-1.93)	9 / 17	0.77 (0.32-1.81)	6 / 17	0.56 (0.21-1.46)	6 / 17	0.57 (0.22-1.50)
Building completion work (45.4)	12 / 13	1.32 (0.58-3.02)	12 / 13	1.37 (0.59-3.17)	11 / 13	1.17 (0.50-2.70)	11 / 13	1.21 (0.52-2.85)
**Wholesale and retail trade; repair of motor vehicles, motorcycles and personal and household goods (50, 51, 52)**	44 / 62	1.18 (0.77-1.80)	43 / 62	1.11 (0.72-1.72	41 / 62	1.10 (0.71-1.69)	40 / 62	1.07 (0.69-1.67)
Sale and repair of motor vehicles (50)	14 / 13	1.79 (0.82-3.90)	14 / 13	1.56 (0.70-3.44)	10 / 13	1.22 (0.52-2.88)	10 / 13	1.14 (0.48-2.72)
Maintenance and repair services of motor vehicles (50.2)	12 / 11	1.80 (0.77-4.22)	12 / 11	1.61 (0.68-3.81)	8 / 11	1.18 (0.46-3.04)	8 / 11	1.18 (0.45-3.08)
Maintenance and repair services of motor vehicles (50.2Z)	12 / 11	1.82 (0.77-4.28)	12 / 11	1.62 (0.68-3.85)	8 / 11	1.15 (0.44-2.97)	8 / 11	1.13 (0.43-2.95)
Wholesale trade and trade intermediaries (51)	8 / 13	0.89 (0.35-2.23)	8 / 13	0.94 (0.37-2.39)	7 / 13	0.81 (0.31-2.11)	7 / 13	0.85 (0.32-2.27)
Retail and repair of household goods (52)	22 / 36	1.04 (0.60-1.82)	21 / 36	0.98 (0.55-1.75)	24 / 36	1.15 (0.67-1.98)	23 / 36	1.12 (0.64-1.97)
Food retailing in specialized stores (52.2)	7 / 10	1.16 (0.43-3.15)	7 / 10	1.21 (0.44-3.32)	7 / 10	1.24 (0.45-3.38)	7 / 10	1.26 (0.46-3.44)
Other retail in specialized stores (52.4)	9 / 12	1.18 (0.48-2.88)	8 / 12	1.01 (0.39-2.59)	11 / 12	1.54 (0.66-3.60)	10 / 12	1.35 (0.56-3.30)
**Hotels and restaurants (55)**	9 / 13	1.30 (0.53-3.16)	9 / 13	1.25 (0.50-3.09)	10 / 13	1.33 (0.57-3.14)	10 / 13	1.24 (0.52-2.96)
Restaurants (55.3)	5 / 9	1.00 (0.32-3.11)	5 / 9	0.98 (0.31-3.08)	5 / 9	0.89 (0.29-2.77)	5 / 9	0.88 (0.28-2.76)
**Transport, storage and communication (60, 61, 62, 63, 64)**	36 / 60	1.11 (0.71-1.73)	36 / 59	1.11 (0.71-1.76)	39 / 59	1.19 (0.77-1.84)	39 / 58	1.19 (0.76-1.86)
Land transport (60)	23 / 35	1.17 (0.67-2.04)	23 / 34	1.27 (0.72-2.24)	27 / 34	1.35 (0.79-2.31)	27 / 33	1.43 (0.82-2.49)
Transport via railways (60.1)	6 / 9	1.19 (0.41-3.47)	6 / 9	1.38 (0.46-4.11)	6 / 9	1.23 (0.42-3.58)	6 / 9	1.46 (0.49-4.35)

Transport via railways (60.1Z)	6 / 9	1.17 (0.40-3.43)	6 / 9	1.34 (0.45-4.01)	6 / 9	1.23 (0.42-3.60)	6 / 9	1.40 (0.47-4.20)
Urban and road transport (60.2)	17 / 25	1.17 (0.61-2.24)	17 / 24	1.25 (0.64-2.46)	20 / 24	1.41 (0.75-2.64)	20 / 23	1.43 (0.75-2.75)
Local road transport of goods (60.2L)	7 / 8	1.46 (0.50-4.25)	7 / 8	1.42 (0.49-4.14)	8 / 8	1.66 (0.59-4.62)	8 / 8	1.43 (0.50-4.10)
Post and telecommunications (64)	8 / 19	0.83 (0.35-1.92)	8 / 19	0.68 (0.28-1.65)	7 / 19	0.75 (0.31-1.84)	7 / 19	0.64 (0.25-1.60)
Post and courier activities (64.1)	5 / 8	1.17 (0.37-3.66)	5 / 8	1.03 (0.31-3.36)	-	-	-	-
National post activities (64.1A)	5 / 8	1.21 (0.38-3.80)	5 / 8	1.08 (0.33-3.52)	-	-	-	-
**Financial intermediation (65, 66, 67)**	10 / 19	0.96 (0.43-2.12)	10 / 17	0.91 (0.40-2.10)	10 / 18	0.98 (0.44-2.19)	10 / 16	0.91 (0.39-2.12)
Financial intermediation (65)	10 / 15	1.22 (0.53-2.80)	10 / 14	1.10 (0.46-2.60)	9 / 14	1.11 (0.47-2.62)	9 / 13	0.98 (0.40-2.41)
Monetary intermediation (65.1)	10 / 15	1.17 (0.51-2.69)	10 / 14	1.03 (0.43-2.46)	9 / 14	1.08 (0.46-2.57)	9 / 13	0.96 (0.39-2.37)
**Real estate, renting and business activities (70, 71, 72, 73, 74)**	18 / 43	0.73 (0.41-1.31)	18 / 42	0.77 (0.42-1.40)	19 / 43	0.79 (0.44-1.41)	19 / 42	0.81 (0.45-1.46)
Services provided primarily to businesses (74)	13 / 24	0.97 (0.48-1.96)	13 / 23	1.03 (0.50-2.13)	13 / 24	0.96 (0.48-1.94)	13 / 23	0.98 (0.48-2.04)
Architectural and engineering activities (74.2)	5 / 19	0.43 (0.16-1.18)	5 / 19	0.39 (0.13-1.13)	6 / 19	0.53 (0.21-1.35)	6 / 19	0.50 (0.18-1.33)
Engineering, technical studies (74.2C)	-	-	-	-	5 / 14	0.57 (0.20-1.65)	5 / 14	0.59 (0.20-1.74)
**Public administration and defence; compulsory social security (75)**	37 / 45	1.50 (0.94-2.39)	37 / 45	1.55 (0.97-2.48)	35 / 45	1.43 (0.90-2.30)	35 / 45	1.48 (0.92-2.38)
General, economic and social administration (75.1)	14 / 14	1.84 (0.86-3.95)	14 / 14	1.85 (0.85-4.01)	14 / 14	1.89 (0.88-4.07)	14 / 14	1.87 (0.87-4.05)
General public administration (75.1A)	10 / 13	1.42 (0.61-3.32)	10 / 13	1.52 (0.64-3.60)	10 / 13	1.41 (0.60-3.29)	10 / 13	1.43 (0.60-3.40)
Public prerogative services (75.2)	23 / 31	1.24 (0.70-2.20)	23 / 31	1.27 (0.71-2.29)	21 / 31	1.16 (0.64-2.09)	21 / 31	1.18 (0.65-2.15)
Defense (75.2C)	13 / 24	0.85 (0.42-1.74)	13 / 24	0.81 (0.39-1.69)	13 / 24	0.88 (0.43-1.79)	13 / 24	0.84 (0.40-1.75)
**Education (80)**	13 / 31	0.83 (0.42-1.63)	12 / 31	0.72 (0.35-1.48)	17 / 31	1.04 (0.56-1.93)	16 / 31	0.89 (0.45-1.73)
Secondary education (80.2)	8 / 11	1.16 (0.45-2.97)	8 / 11	0.99 (0.37-2.70)	10 / 11	1.46 (0.60-3.54)	10 / 11	1.25 (0.49-3.19)
**Health and social work (85)**	15 / 41	0.62 (0.33-1.15)	15 / 41	0.64 (0.34-1.20)	16 / 41	0.65 (0.35-1.20)	16 / 41	0.66 (0.36-1.24)
Activities for human health (85.1)	13 / 32	0.64 (0.33-1.27)	13 / 32	0.65 (0.33-1.32)	15 / 32	0.76 (0.40-1.45)	15 / 32	0.76 (0.39-1.48)
Hospital activities (85.1A)	6 / 12	0.98 (0.35-2.74)	6 / 12	1.04 (0.36-3.03)	7 / 12	1.14 (0.43-3.05)	7 / 12	1.24 (0.45-3.40)
Medical practice (85.1C)	-	-	-	-	6 / 13	0.65 (0.24-1.78)	6 / 13	0.65 (0.23-1.82)
**Other community, social and personal services activities (90, 91, 92, 93)**	7 / 13	1.15 (0.45-2.98)	7 / 13	1.21 (0.46-3.18)	6 / 13	0.96 (0.35-2.58)	6 / 13	1.05 (0.38-2.88)

*Adjusted for sibship size, born from multiple pregnancy, personal history of testicular trauma, family history of testicular cancer and family history of cryptorchidism
