# Supplementary material for: Parental occupations at birth and risk of adult testicular germ cell tumors in offspring: a French nationwide case–control study
Source: Front Public Health. 2024 Jan 16;11:1303998. doi: 10.3389/fpubh.2023.1303998 (PMC10825020; doi:10.3389/fpubh.2023.1303998)
Supplement: Supplementary file 4 [file Data_Sheet_4.pdf]

Supplementary material

Table S4. Odds ratios (OR) and 95% confidence intervals (CI) for TGCT associated with mother's job (ISCO-1968) and industry sector (NAF-1999) at birth, overall, sensitivity analyses, case-control study, N=1124, France, 2015-2018.

	**Excluding TGCT cases with personal history of cryptorchidism (N=1084)**				**Excluding TGCT cases not confirmed by pathology report (N=1081)**			
	**N cases / N controls**	**Crude OR (95% CI)**	**N cases / N controls**	**Adjusted OR* (95% CI)**	**N cases / N controls**	**Crude OR (95% CI)**	**N cases / N controls**	**Adjusted OR* (95% CI)**
**ISCO-1968 CODES**								
**Professional, Technical and Related Workers (0/1)**	78 / 515	0.97 (0.70-1.33)	77 / 137	0.94 (0.68-1.31)	84 / 137	1.09 (0.80-1.50)	83 / 137	1.06 (0.77-1.48)
Medical, dental, veterinary and related workers (0-6/0-7)	23 / 50	0.72 (0.43-1.22)	22 / 50	0.69 (0.41-1.19)	23 / 50	0.77 (0.45-1.29)	22 / 50	0.75 (0.44-1.28)
Medical doctors (0-61)	5 / 7	1.08 (0.33-3.58)	5 / 7	1.22 (0.36-4.12)	5 / 7	1.22 (0.37-4.01)	5 / 7	1.40 (0.42-4.72)
Professional Nurses (0-71)	13 / 24	0.90 (0.45-1.80)	12 / 24	0.82 (0.40-1.71)	13 / 24	0.96 (0.48-1.93)	12 / 24	0.92 (0.44-1.91)
Professional Nurse (General) (0-71.10)	5 / 15	0.57 (0.20-1.60)	5 / 15	0.62 (0.22-1.75)	6 / 15	0.73 (0.28-1.94)	6 / 15	0.84 (0.32-2.24)
Accountants (1-1)	12 / 13	1.76 (0.79-3.95)	12 / 13	1.71 (0.76-3.83)	14 / 13	2.17 (1.00-4.73)	14 / 13	2.10 (0.96-4.58)
Accountants (1-10)	12 / 13	1.76 (0.79-3.95)	12 / 13	1.70 (0.76-3.82)	14 / 13	2.17 (0.99-4.72)	14 / 13	2.09 (0.96-4.57)
Auditor (1-10.20)	12 / 11	2.09 (0.90-4.84)	12 / 11	2.03 (0.87-4.71)	14 / 11	**2.56 (1.14-5.79)**	14 / 11	**2.51 (1.11-5.70)**
Teachers (1-3)	29 / 41	1.20 (0.72-1.98)	29 / 41	1.20 (0.72-2.02)	31 / 41	1.28 (0.78-2.10)	31 / 41	1.26 (0.75-2.11)
Secondary education teachers (1-32)	17 / 14	**2.34 (1.12-4.87)**	17 / 14	**2.30 (1.09-4.88)**	18 / 14	**2.39 (1.15-4.95)**	18 / 14	**2.36 (1.11-5.03)**
Primary Education Teachers (1-33)	9 / 19	0.76 (0.33-1.72)	9 / 19	0.78 (0.34-1.80)	10 / 19	0.83 (0.38-1.84)	10 / 19	0.81 (0.36-1.85)
First-Level Education Teacher (1-33.20)	9 / 19	0.79 (0.35-1.80)	9 / 19	0.81 (0.35-1.89)	10 / 19	0.87 (0.39-1.92)	10 / 19	0.84 (0.37-1.94)
Professional, technical and related workers not elsewhere classified (1-9)	6 / 17	0.70 (0.27-1.81)	6 / 17	0.66 (0.25-1.77)	7 / 17	0.82 (0.33-2.02)	7 / 17	0.78 (0.31-1.97)
Social workers (1-93)	-	-	-	-	5 / 7	1.54 (0.48-5.01)	5 / 7	1.26 (0.37-4.23)
**Administrative and Managerial Workers (2)**	5 / 11	0.62 (0.20-1.90)	5 / 11	0.60 (0.19-1.86)	5 / 11	0.73 (0.24-2.21)	5 / 11	0.68 (0.22-2.09)
Managers (2-1)	5 / 11	0.62 (0.20-1.90)	5 / 11	0.60 (0.19-1.86)	5 / 11	0.73 (0.24-2.21)	5 / 11	0.68 (0.22-2.09)
Managers not elsewhere classified (2-19)	5 / 9	0.69 (0.21-2.24)	5 / 9	0.68 (0.21-2.24)	5 / 9	0.84 (0.27-2.66)	5 / 9	0.83 (0.26-2.66)
**Clerical and Related Workers (3)**	90 / 125	1.31 (0.95-1.79)	89 / 123	1.28 (0.92-1.77)	85 / 125	1.15 (0.84-1.58)	84 / 123	1.13 (0.81-1.58)
Stenographers, typists and card-and tape-punching machine operators (3-2)	27 / 45	1.11 (0.67-1.84)	27 / 45	1.06 (0.63-1.78)	25 / 45	0.94 (0.56-1.58)	25 / 45	0.88 (0.52-1.50)

Stenographers, Typists and Teletypists (3-21)	27 / 45	1.11 (0.67-1.84)	27 / 45	1.06 (0.63-1.78)	25 / 45	0.94 (0.56-1.58)	25 / 45	0.88 (0.52-1.50)
Stenographer-Typist (General) (3-21.10)	18 / 32	1.01 (0.55-1.86)	18 / 32	0.96 (0.51-1.79)	17 / 32	0.88 (0.47-1.63)	17 / 32	0.83 (0.44-1.58)
Stenographic Secretary (3-21.20)	9 / 13	1.37 (0.58-3.26)	9 / 13	1.33 (0.55-3.21)	8 / 13	1.13 (0.46-2.77)	8 / 13	1.02 (0.40-2.58)
Bookkeepers, cashiers and related workers (3-3)	7 / 19	0.57 (0.23-1.39)	7 / 18	0.60 (0.24-1.50)	6 / 19	0.47 (0.18-1.21)	6 / 18	0.53 (0.20-1.38)
Bookkeepers and cashiers (3-31)	6 / 13	0.69 (0.25-1.87)	6 / 12	0.77 (0.28-2.18)	5 / 13	0.55 (0.19-1.58)	5 / 12	0.67 (0.23-2.01)
Clerical and related workers not elsewhere classified (3-9)	42 / 49	1.43 (0.92-2.23)	41 / 49	1.34 (0.85-2.12)	41 / 49	1.37 (0.88-2.14)	40 / 49	1.33 (0.83-2.11)
Correspondence and reporting clerks (3-93)	34 / 41	1.47 (0.91-2.38)	34 / 41	1.39 (0.85-2.29)	34 / 41	1.44 (0.89-2.32)	34 / 41	1.39 (0.84-2.29)
Office clerk (general) (3-93.10)	29 / 35	1.57 (0.93-2.64)	29 / 35	1.47 (0.85-2.53)	29 / 35	1.53 (0.91-2.57)	29 / 35	1.47 (0.86-2.54)
**Sales Workers (4)**	19 / 35	0.92 (0.51-1.65)	19 / 35	0.91 (0.50-1.65)	20 / 35	1.00 (0.56-1.77)	20 / 35	1.01 (0.56-1.82)
Salesmen, shop assistants and related workers (4-5)	12 / 19	1.06 (0.50-2.27)	12 / 19	1.03 (0.48-2.22)	11 / 19	1.03 (0.48-2.21)	11 / 19	1.01 (0.46-2.21)
Salesmen, shop assistants and demonstrators (4-51)	12 / 18	1.10 (0.51-2.37)	12 / 18	1.06 (0.49-2.31)	11 / 18	1.07 (0.49-2.32)	11 / 18	1.05 (0.47-2.31)
Retail trade salesman (4-51.30)	7 / 15	0.70 (0.27-1.78)	7 / 15	0.72 (0.28-1.88)	6 / 15	0.68 (0.26-1.79)	6 / 15	0.73 (0.27-1.94)
**Service Workers (5)**	44 / 75	0.93 (0.62-1.40)	44 / 73	0.97 (0.64-1.48)	41 / 75	0.86 (0.57-1.30)	41 / 73	0.89 (0.58-1.36)
Cooks, waiters, bartenders and related workers (5-3)	6 / 12	0.72 (0.25-2.03)	6 / 12	0.85 (0.29-2.46)	6 / 12	0.67 (0.24-1.86)	6 / 12	0.81 (0.28-2.30)
Maids and related housekeeping service workers not elsewhere classified (5-4)	7 / 6	1.64 (0.53-5.11)	7 / 6	1.74 (0.55-5.51)	8 / 6	1.99 (0.67-5.95)	8 / 6	2.02 (0.65-6.28)
Maids and related housekeeping service workers not elsewhere classified (5-40)	7 / 6	1.62 (0.52-5.07)	7 / 6	1.72 (0.54-5.45)	8 / 6	1.97 (0.66-5.90)	8 / 6	1.99 (0.64-6.20)
Building caretakers, charworkers, cleaners and related workers (5-5)	11 / 21	0.73 (0.34-1.55)	11 / 21	0.68 (0.32-1.47)	10 / 21	0.69 (0.31-1.49)	10 / 21	0.65 (0.29-1.44)
Charworkers, cleaners and related workers (5-52)	11 / 20	0.76 (0.35-1.63)	11 / 20	0.72 (0.33-1.57)	10 / 20	0.71 (0.32-1.55)	10 / 20	0.69 (0.31-1.54)
Charworker (5-52.20)	11 / 20	0.78 (0.36-1.67)	11 / 20	0.73 (0.34-1.59)	10 / 20	0.71 (0.32-1.56)	10 / 20	0.69 (0.31-1.54)
Service workers not elsewhere classified (5-9)	13 / 22	1.15 (0.57-2.33)	13 / 20	1.29 (0.62-2.68)	10 / 22	0.88 (0.41-1.90)	10 / 20	0.92 (0.41-2.05)
Other service workers (5-99)	13 / 22	1.15 (0.57-2.32)	13 / 20	1.29 (0.62-2.68)	10 / 22	0.88 (0.41-1.90)	10 / 20	0.92 (0.41-2.05)
Nursing Aid (5-99.40)	12 / 21	1.14 (0.55-2.35)	12 / 19	1.26 (0.59-2.69)	10 / 21	0.93 (0.43-2.02)	10 / 19	0.97 (0.43-2.17)
**Agricultural, Animal Husbandry and Forestry Workers, fishermen and hunters (6)**	10 / 14	1.18 (0.51-2.73)	9 / 14	1.09 (0.45-2.66)	10 / 14	1.05 (0.45-2.44)	9 / 14	0.99 (0.41-2.39)
Farmers (6-1)	9 / 10	1.48 (0.59-3.74)	8 / 10	1.35 (0.51-3.60)	9 / 10	1.36 (0.54-3.43)	8 / 10	1.26 (0.47-3.35)
Specialized farmers (6-12)	6 / 7	1.30 (0.43-3.99)	5 / 7	1.16 (0.34-3.93)	6 / 7	1.24 (0.40-3.79)	5 / 7	1.11 (0.33-3.77)

**Production and Related Workers, Transport Equipment operators and labourers (7/8/9)**	17 / 46	0.59 (0.32-1.06)	17 / 44	0.62 (0.34-1.13)	16 / 46	0.56 (0.31-1.01)	16 / 44	0.57 (0.31-1.06)
Tailors, dressmakers, sewers, upholsterers and related workers (7-9)	-	-	-	-	5 / 12	0.62 (0.21-1.82)	5 / 12	0.67 (0.22-1.99)
**NAF-1999 CODES**								
**Agriculture, hunting and forestry (01, 02)**	10 / 14	1.14 (0.49-2.66)	9 / 14	1.04 (0.42-2.54)	10 / 14	1.01 (0.43-2.35)	9 / 14	0.93 (0.38-2.27)
Agriculture, hunting and related service activities (01)	10 / 14	1.14 (0.49-2.66)	9 / 14	1.04 (0.42-2.54)	10 / 14	1.01 (0.43-2.35)	9 / 14	0.93 (0.38-2.27)
Farming (01.2)	5 / 6	1.24 (0.37-4.19)	-	-	5 / 6	1.14 (0.34-3.85)	-	-
**Manufacturing (15 to 37)**	18 / 43	0.66 (0.37-1.18)	18 / 43	0.66 (0.36-1.20)	18 / 43	0.69 (0.39-1.23)	18 / 43	0.69 (0.38-1.25)
Food industry (15)	5 / 8	0.90 (0.28-2.87)	5 / 8	0.86 (0.26-2.88)	-	-	-	-
Clothing and fur industry (18)	-	-	-	-	6 / 10	0.93 (0.33-2.67)	6 / 10	0.99 (0.34-2.89)
Manufacture of textile clothing (18.2)	-	-	-	-	6 / 10	1.00 (0.35-2.84)	6 / 10	1.11 (0.38-3.22)
**Wholsale and retail rade; repair of motor vehicls, motorcycles and personal and household goods (50, 51, 52)**	22 / 44	0.85 (0.50-1.47)	22 / 43	0.85 (0.49-1.49)	22 / 44	0.83 (0.48-1.43)	22 / 43	0.88 (0.51-1.53)
Retail and repair of household goods (52)	19 / 40	0.79 (0.44-1.41)	19 / 39	0.82 (0.45-1.48)	19 / 40	0.77 (0.43-1.36)	19 / 39	0.85 (0.47-1.54)
Retail trade in non-specialized stores (52.1)	5 / 10	0.84 (0.27-2.59)	5 / 9	0.96 (0.30-3.11)	-	-	-	-
Supermarkets (52.1D)	5 / 9	0.90 (0.28-2.94)	5 / 8	1.07 (0.30-3.75)	-	-	-	-
Other retail in specialized stores (52.4)	7 / 11	0.97 (0.36-2.60)	7 / 11	0.93 (0.34-2.52)	6 / 11	0.89 (0.32-2.49)	6 / 11	0.87 (0.31-2.45)
**Hotels and restaurants (55)**	10 / 19	0.76 (0.34-1.70)	10 / 19	0.80 (0.35-1.83)	10 / 19	0.74 (0.33-1.63)	10 / 19	0.79 (0.35-1.78)
Restaurants (55.3)	7 / 12	0.93 (0.35-2.47)	7 / 12	1.02 (0.37-2.79)	7 / 12	0.88 (0.34-2.30)	7 / 12	0.95 (0.35-2.58)
**Transport, storage and communication (60, 61, 62, 63, 64)**	8 / 14	0.83 (0.34-2.07)	8 / 13	0.87 (0.34-2.24)	8 / 14	0.88 (0.35-2.19)	8 / 13	0.90 (0.35-2.32)
**Financial intermediation (65, 66, 67)**	6 / 15	0.67 (0.25-1.75)	6 / 15	0.65 (0.25-1.73)	6 / 15	0.65 (0.25-1.72)	6 / 15	0.62 (0.24-1.65)
**Real estate, renting and business activities (70, 71, 72, 73, 74)**	12 / 19	1.07 (0.50-2.27)	12 / 18	1.10 (0.50-2.39)	15 / 19	1.35 (0.66-2.75)	15 / 18	1.33 (0.63-2.79)
Services provided primarily to businesses (74)	6 / 11	0.96 (0.35-2.68)	6 / 11	0.90 (0.32-2.58)	9 / 11	1.42 (0.57-3.54)	9 / 11	1.23 (0.48-3.19)
Legal, accounting and management consulting activities (74.1)	5 / 7	1.33 (0.41-4.29)	5 / 7	1.13 (0.34-3.81)	7 / 7	2.00 (0.68-5.86)	7 / 7	1.63 (0.53-5.02)
**Public administration and defence; compulsory social security (75)**	17 / 34	0.83 (0.45-1.54)	17 / 33	0.99 (0.52-1.87)	16 / 34	0.75 (0.41-1.40)	16 / 33	0.92 (0.48-1.76)
General, economic and social administration (75.1)	11 / 23	0.88 (0.41-1.86)	11 / 22	0.99 (0.45-2.18)	10 / 23	0.76 (0.35-1.64)	10 / 22	0.88 (0.39-1.97)
General public administration (75.1A)	9 / 21	0.75 (0.33-1.70)	9 / 20	0.82 (0.35-1.93)	8 / 21	0.66 (0.28-1.53)	8 / 20	0.72 (0.30-1.77)
**Education (80)**	33 / 47	1.14 (0.71-1.84)	33 / 47	1.16 (0.71-1.90)	35 / 47	1.22 (0.76-1.95)	35 / 47	1.22 (0.75-1.98)

Primary education (80.1)	13 / 22	0.87 (0.42-1.77)	13 / 22	0.88 (0.42-1.82)	14 / 22	0.94 (0.47-1.89)	14 / 22	0.91 (0.44-1.88)
Primary education (80.1Z)	13 / 22	0.80 (0.39-1.67)	13 / 22	0.82 (0.39-1.72)	14 / 22	0.88 (0.43-1.78)	14 / 22	0.85 (0.41-1.78)
Secondary education (80.2)	17 / 14	**2.31 (1.10-4.85)**	17 / 14	**2.22 (1.05-4.71)**	18 / 14	**2.39 (1.15-4.97)**	18 / 14	**2.27 (1.07-4.83)**
General secondary education (80.2A)	8 / 6	1.89 (0.61-5.91)	8 / 6	1.59 (0.50-5.03)	8 / 6	1.79 (0.57-5.60)	8 / 6	1.45 (0.45-4.64)
**Health and social work (85)**	52 / 93	0.93 (0.64-1.35)	50 / 91	0.89 (0.60-1.31)	49 / 93	0.92 (0.62-1.34)	47 / 91	0.86 (0.58-1.28)
Activities for human health (85.1)	26 / 64	0.68 (0.42-1.10)	26 / 62	0.70 (0.42-1.14)	26 / 64	0.71 (0.44-1.16)	26 / 62	0.74 (0.45-1.21)
Hospital activities (85.1A)	19 / 37	0.86 (0.48-1.55)	19 / 36	0.86 (0.47-1.57)	19 / 37	0.90 (0.50-1.62)	19 / 36	0.90 (0.49-1.65)
Social action (85.3)	11 / 16	1.12 (0.50-2.52)	11 / 16	1.14 (0.50-2.59)	10 / 16	1.09 (0.48-2.47)	10 / 16	1.06 (0.45-2.45)
**Other community, social and personal services activities (90, 91, 92, 93)**	8 / 14	0.92 (0.37-2.29)	8 / 14	0.94 (0.37-2.38)	8 / 14	0.94 (0.38-2.31)	8 / 14	0.97 (0.38-2.45)
Recreational, cultural and sporting activities (92)	5 / 10	0.70 (0.23-2.10)	5 / 10	0.60 (0.19-1.83)	-	-	-	-
**Private households with employed persons (95)**	5 / 10	0.70 (0.23-2.10)	5 / 10	0.60 (0.19-1.83)	-	-	-	-
Domestic services (95.0)	5 / 10	0.73 (0.24-2.18)	5 / 10	0.62 (0.20-1.91)	-	-	-	-
Domestic services (95.0Z)	5 / 10	0.71 (0.23-2.16)	5 / 10	0.61 (0.20-1.88)	-	-	-	-

*Adjusted for sibship size, born from multiple pregnancy, personal history of testicular trauma, family history of testicular cancer and family history of cryptorchidism
