# Supplementary material for: Parental occupations at birth and risk of adult testicular germ cell tumors in offspring: a French nationwide case–control study
Source: Front Public Health. 2024 Jan 16;11:1303998. doi: 10.3389/fpubh.2023.1303998 (PMC10825020; doi:10.3389/fpubh.2023.1303998)
Supplement: Supplementary file 5 [file Data_Sheet_5.pdf]

Supplementary material

Table S5. Odds ratios (OR) and 95% confidence intervals (CI) for TGCT associated with father's job (ISCO-1968) and industry sector (NAF-1999) at birth dixit interviewed mothers, overall, case-control study, France, 2015-2018.

	N cases / N controls	Crude OR (95% CI)	N cases / N controls	Adjusted OR* (95% CI)
**ISCO-1968 CODES**				
**Professional, Technical and Related Workers (0/1)**	63/93	0.68 (0.45-1.01)	62/93	0.69 (0.45-1.04)
Architects, engineers and related technicians (0-2/0-3)	19/30	0.64 (0.34-1.21)	19/30	0.69 (0.36-1.30)
Medical, dental, veterinary and related workers (0-6/0-7)	7/17	0.40 (0.16-1.01)	7/17	0.41 (0.16-1.04)
Medical doctors (0-61)	6/10	0.66 (0.22-1.96)	6/10	0.67 (0.23-2.02)
Specialized physician (0-61.20)	5/5	1.04 (0.27-3.95)	5/5	0.87 (0.23-3.28)
Teachers (1-3)	14/22	0.65 (0.32-1.35)	13/22	0.62 (0.29-1.35)
Secondary education teachers (1-32)	8/10	0.88 (0.32-2.39)	8/10	0.93 (0.32-2.71)
**Administrative and managerial workers (2)**	18/15	1.40 (0.66-2.96)	18/15	1.40 (0.64-3.04)
Managers (2-1)	18/15	1.40 (0.66-2.96)	18/15	1.40 (0.64-3.04)
Managers not elsewhere classified (2-19)	11/12	1.17 (0.49-2.82)	11/12	1.14 (0.46-2.80)
Other managers (2-19.90)	10/9	1.49 (0.57-3.87)	10/9	1.47 (0.55-3.93)
**Clerical and related workers (3)**	24/27	0.88 (0.47-1.64)	24/27	0.85 (0.45-1.61)
Bookkeepers, cashiers and related workers (3-3)	9/6	1.45 (0.48-4.34)	9/6	1.12 (0.37-3.44)
Clerical and related workers not elsewhere classified (3-9)	10/12	0.81 (0.32-2.06)	10/12	0.80 (0.31-2.05)
Correspondence and reporting clerks (3-93)	6/8	0.77 (0.24-2.50)	6/8	0.72 (0.22-2.39)
Office clerk (general) (3-93.10)	5/6	1.10 (0.30-4.08)	5/6	1.00 (0.27-3.77)
**Sales Workers (4)**	11/13	0.68 (0.28-1.66)	11/13	0.78 (0.31-1.97)
Technical salesmen, commercial travellers and manufacturers' agents (4-3)	6/6	0.78 (0.22-2.83)	6/6	0.89 (0.23-3.49)
Technical salesmen and service advisers (4-31)	6/6	0.79 (0.22-2.87)	6/6	0.89 (0.23-3.50)
Technical salesman (4-31.20)	6/6	0.85 (0.23-3.10)	6/6	0.89 (0.23-3.50)
**Service Workers (5)**	22/11	2.03 (0.92-4.49)	22/11	2.03 (0.89-4.60)
**Agricultural, animal husbandry and forestry workers, fishermen and hunters (6)**	21/14	1.37 (0.66-2.84)	20/14	1.41 (0.65-3.06)
Farmers (6-1)	13/6	2.15 (0.77-5.97)	12/6	2.43 (0.83-7.14)
Specialized farmers (6-12)	5/5	1.26 (0.34-4.63)	5/5	1.63 (0.43-6.17)

**Production and Related Workers, Transport Equipment operators and labourers (7/8/9)**	90/75	1.08 (0.72-1.62)	89/75	1.03 (0.68-1.56)
Blacksmiths, toolmakers and machine-tool operators (8-3)	15/5	2.88 (0.98-8.46)	15/5	2.54 (0.85-7.61)
Machinery fitters, machine assemblers and precision-instrument makers [except electrical] (8-4)	13/10	1.03 (0.42-2.53)	13/10	1.00 (0.40-2.53)
Motor-vehicle mechanics (8-43)	6/5	0.91 (0.25-3.36)	6/5	0.81 (0.22-3.03)
Machinery fitters, machine assemblers and precision-instrument makers [except electrical] not elsewhere classified (8-49)	5/5	0.98 (0.27-3.63)	5/5	1.06 (0.28-4.08)
Electrical fitters and related electrical and electronics workers (8-5)	5/12	0.38 (0.13-1.15)	5/12	0.37 (0.12-1.15)
Plumbers, welders, sheet-metal and structural metal preparers and erectors (8-7)	5/5	0.53 (0.14-2.08)	-	-
Bricklayers, carpenters and other construction workers (9-5)	14/6	1.99 (0.70-5.62)	14/6	2.30 (0.78-6.74)
Material handling and related equipment operators, dockers and freight handlers (9-7)	6/5	1.20 (0.35-4.08)	6/5	1.59 (0.42-5.94)
Transport equipment operators (9-8)	11/13	0.97 (0.41-2.30)	11/13	1.00 (0.42-2.39)
Motor-vehicle drivers (9-85)	9/11	0.80 (0.31-2.03)	9/11	0.82 (0.32-2.09)
**NAF-1999 CODES**				
**Agriculture, hunting and forestry (01, 02)**	24/14	1.75 (0.86-3.58)	23/14	1.79 (0.85-3.19)
Agriculture, hunting and related service activities (01)	21/11	2.00 (0.92-4.35)	20/11	2.18 (0.96-4.92)
Crops (01.1)	11/5	2.73 (0.90-8.33)	10/5	**3.29 (1.04-10.44)**
**Manufacturing (15 to 37)**	52/54	0.91 (0.58-1.45)	51/54	0.92 (0.57-1.48)
Food industry (15)	7/7	1.05 (0.34-3.25)	7/7	1.16 (0.37-3.64)
**Construction (45)**	31/23	1.13 (0.62-2.08)	31/23	1.10 (0.59-2.07)
Construction of building or civil engineering works (45.2)	9/6	1.20 (0.39-3.64)	9/6	1.42 (0.44-4.62)
Installation works (45.3)	9/7	1.20 (0.41-3.51)	9/7	1.12 (0.37-3.37)
Building completion work (45.4)	10/7	1.19 (0.41-3.40)	10/7	1.10 (0.38-3.18)
**Wholesale and retail trade ; repair of motor vehicles,motorcycles and personal and household goods (50, 51, 52)**	21/23	0.79 (0.41-1.51)	21/23	0.86 (0.44-1.66)
Wholesale trade and trade intermediaries (51)	9/9	0.92 (0.35-2.43)	9/9	1.10 (0.40-2.97)
Retail and repair of household goods (52)	8/8	0.92 (0.3-2.60)	8/8	0.89 (0.31-2.57)
**Hotels and restaurants (55)**	6/5	1.19 (0.34-4.17)	6/5	1.06 (0.29-3.85)
**Transport, storage and communication (60, 61, 62, 63, 64)**	26/26	1.18 (0.64-2.15)	26/26	1.18 (0.63-2.20)
Land transport (60)	16/15	1.21 (0.56-2.59)	16/15	1.20 (0.55-2.64)
Transport via railways (60.1)	8/5	2.36 (0.67-8.31)	8/5	2.30 (0.62-8.51)
Transport via railways (60.1Z)	8/5	2.42 (0.69-8.46)	8/5	2.34 (0.65-8.48)
Urban and road transport (60.2)	8/10	0.76 (0.29-2.02)	8/10	0.78 (0.29-2.12)
Post and telecommunications (64)	5/7	0.91 (0.27-3.13)	5/7	0.92 (0.26-3.25)

**Financial intermediation (65, 66, 67)**	10/8	1.19 (0.42-3.34)	10/8	0.96 (0.34-2.72)
Financial intermediation (65)	9/7	1.30 (0.44-3.91)	9/7	1.02 (0.34-3.11)
Monetary intermediation (65.1)	9/7	1.22 (0.41-3.65)	9/7	0.96 (0.32-2.92)
**Real estate, renting and business activities (70, 71, 72, 73, 74)**	18/21	0.98 (0.49-1.97)	18/21	0.92 (0.46-1.87)
Computer and related activities (72)	5/6	0.68 (0.19-2.42)	5/6	0.64 (0.17-2.37)
Services provided primarily to businesses (74)	10/10	1.29 (0.51-3.27)	10/10	1.25 (0.49-3.22)
Legal, accounting and management consulting activities (74.1)	5/6	1.09 (0.32-3.79)	5/6	1.12 (0.32-3.93)
**Public administration and defence; compulsory social security (75)**	29/23	1.24 (0.67-2.29)	29/23	1.36 (0.72-2.57)
General, economic and social administration (75.1)	12/7	1.79 (0.66-4.88)	12/7	1.74 (0.64-4.74)
General public administration (75.1A)	9/5	2.07 (0.64-6.74)	9/5	1.91 (0.59-6.18)
Public prerogative services (75.2)	17/15	1.01 (0.47-2.21)	17/15	1.20 (0.53-2.69)
Defense (75.2C)	8/12	0.62 (0.23-1.64)	8/12	0.70 (0.26-1.91)
**Education (80)**	15/24	0.54 (0.27-1.09)	14/24	0.50 (0.23-1.07)
Secondary education (80.2)	8/12	0.59 (0.22-1.56)	8/12	0.56 (0.20-1.58)
General secondary education (80.2A)	5/6	0.49 (0.13-1.89)	5/6	0.10 (0.09-1.80)
**Health and social work (85)**	12/17	0.73 (0.33-1.62)	12/17	0.75 (0.33-1.69)
Activities for human health (85.1)	9/16	0.54 (0.22-1.28)	9/16	0.55 (0.23-1.33)

*Adjusted for sibship size, born from multiple pregnancy, personal history of testicular trauma, family history of testicular cancer and family history of cryptorchidism
