# Supplementary material for: Parental occupations at birth and risk of adult testicular germ cell tumors in offspring: a French nationwide case–control study
Source: Front Public Health. 2024 Jan 16;11:1303998. doi: 10.3389/fpubh.2023.1303998 (PMC10825020; doi:10.3389/fpubh.2023.1303998)
Supplement: Supplementary file 6 [file Data_Sheet_6.pdf]

Supplementary material

Table S6. Odds ratios (OR) and 95% confidence intervals (CI) for TGCT associated with mother's job (ISCO-1968) and industry sector (NAF-1999) at birth dixit interviewed mothers, overall, case-control study, France, 2015-2018.

	N cases / N controls	Crude OR (95% CI)	N cases / N controls	Adjusted OR* (95% CI)
**ISCO-1968 CODES**				
**Professional, Technical and Related Workers (0/1)**	54/68	0.88 (0.57-1.36)	53/68	0.91 (0.58-1.44)
Medical, dental, veterinary and related workers (0-6/0-7)	17/24	0.78 (0.40-1.55)	16/24	0.81 (0.40-1.64)
Professional Nurses (0-71)	13/11	1.32 (0.56-3.12)	12/11	1.29 (0.53-3.14)
Professional Nurse (General) (0-71.10)	12/9	1.50 (0.59-3.79)	11/9	1.45 (0.56-3.80)
Accountants (1-1)	5/5	1.38 (0.34-5.61)	5/5	1.47 (0.36-6.03)
Accountants (1-10)	5/5	1.38 (0.34-5.61)	5/5	1.47 (0.36-6.03)
Teachers (1-3)	26/26	1.15 (0.63-2.09)	26/26	1.27 (0.68-2.37)
Secondary education teachers (1-32)	11/10	1.54 (0.62-3.81)	11/10	2.02 (0.76-5.34)
Primary Education Teachers (1-33)	13/12	1.07 (0.46-2.46)	13/12	1.02 (0.44-2.39)
First-Level Education Teacher (1-33.20)	12/12	0.96 (0.41-2.27)	12/12	0.92 (0.38-2.21)
**Administrative and Managerial Workers (2)**	8/6	1.45 (0.46-4.59)	8/6	1.29 (0.40-4.18)
Managers (2-1)	8/6	1.45 (0.46-4.59)	8/6	1.29 (0.40-4.18)
Managers not elsewhere classified (2-19)	7/5	1.63 (0.47-5.61)	7/5	1.49 (0.42-5.27)
**Clerical and Related Workers (3)**	61/51	1.18 (0.75-1.84)	61/51	1.17 (0.74-1.87)
Stenographers, typists and card-and tape-punching machine operators (3-2)	15/12	1.18 (0.52-2.70)	15/12	1.16 (0.49-2.73)
Stenographers, Typists and Teletypists (3-21)	15/12	1.18 (0.52-2.70)	15/11	1.16 (0.49-2.73)
Stenographer-Typist (General) (3-21.10)	15/12	1.19 (0.52-2.73)	15/12	1.17 (0.50-2.77)
Bookkeepers, cashiers and related workers (3-3)	9/10	0.92 (0.35-2.40)	9/10	0.78 (0.30-2.06)
Bookkeepers and cashiers (3-31)	7/7	1.06 (0.35-3.24)	7/7	0.94 (0.31-2.90)

Clerical and related workers not elsewhere classified (3-9)	31/25	1.20 (0.66-2.17)	31/25	1.22 (0.66-2.25)
Correspondence and reporting clerks (3-93)	26/20	1.34 (0.70-2.58)	26/10	1.43 (0.72-2.81)
Office clerk (general) (3-93.10)	21/17	1.20 (0.58-2.46)	21/17	1.22 (0.57-2.58)
**Sales Workers (4)**	14/8	1.41 (0.54-3.63)	14/8	1.51 (0.57-4.01)
**Service Workers (5)**	30/24	1.31 (0.72-2.38)	30/23	1.48 (0.79-2.76)
Building caretakers, charworkers, cleaners and related workers (5-5)	5/7	0.59 (0.17-2.08)	5/6	0.66 (0.17-2.53)
Charworkers, cleaners and related workers (5-52)	5/7	0.59 (0.17-2.08)	5/6	0.66 (0.17-253)
Charworker (5-52.20)	5/6	0.67 (0.18-2.48)	5/5	0.77 (0.19-3.13)
Service workers not elsewhere classified (5-9)	11/10	1.47 (0.61-3.56)	11/10	1.58 (0.64-3.92)
Other service workers (5-99)	11/10	1.47 (0.61-3.56)	11/10	1.58 (0.64-3.92)
Nursing Aid (5-99.40)	9/9	1.25 (0.48-3.25)	9/9	1.33 (0.50-3.58)
**Agricultural, Animal Husbandry and Forestry Workers, fishermen and hunters (6)**	8/5	1.32 (0.40-4.31)	7/5	1.15 (0.31-4.27)
**Production and Related Workers, Transport Equipment operators and labourers (7/8/9)**	16/14	0.82 (0.37-1.85)	15/14	0.75 (0.32-1.76)
**NAF-1999 CODES**				
**Agriculture, hunting and forestry (01, 02)**	10/5	1.99 (0.64-6.20)	9/5	2.14 (0.61-7.45)
Agriculture, hunting and related service activities (01)	10/5	1.99 (0.64-6.20)	9/5	2.14 (0.61-7.45)
**Manufacturing (15 to 37)**	27/25	0.90 (0.48-1.66)	26/15	0.91 (0.47-1.73)
**Wholsale and retail rade; repair of motor vehicls, motorcycles and personal and household goods (50, 51, 52)**	19/14	1.14 (0.53-2.45)	19/14	1.14 (0.52-2.49)
Wholesale trade and commission trade, except of motor vehicles and motorcycles (51)	5/5	0.96 (0.24-3.78)	5/5	0.97 (0.24-3.95)
Retail and repair of household goods (52)	13/9	1.08 (0.43-2.73)	13/9	1.05 (0.41-2.69)
Other retail in specialized stores (52.4)	6/5	1.04 (0.29-3.70)	6/5	1.04 (0.28-3.85)
**Transport, storage and communication (60, 61, 62, 63, 64)**	10/10	0.88 (0.34-2.29)	10/10	0.76 (0.29-2.02)
**Financial intermediation (65,66,67)**	10/10	1.26 (0.49-3.23)	10/10	1.22 (0.47-3.18)
**Real estate, renting and business activities (70, 71, 72, 73, 74)**	10/9	1.09 (0.41-2.89)	10/9	1.03 (0.38-2.80)
Services provided primarily to businesses (74)	5/5	0.85 (0.24-3.07)	5/5	0.81 (0.21-3.03)
**Public administration and defence; compulsory social security (75)**	13/16	0.74 (0.33-1.65)	13/16	0.73 (0.32-1.70)
General, economic and social administration (75.1)	7/9	0.87 (0.30-2.51)	7/9	0.90 (0.29-2.83)

General public administration (75.1A)	6/9	0.77 (0.25-2.31)	6/9	0.79 (0.24-2.58)
**Education (80)**	30/30	1.15 (0.65-2.02)	30/30	1.27 (0.71-2.29)
Primary education (80.1)	13/13	0.98 (0.43-2.23)	13/13	0.96 (0.42-2.21)
Primary education (80.1Z)	13/12	1.03 (0.45-2.37)	13/12	1.00 (0.43-2.33)
Secondary education (80.2)	11/11	1.51 (0.62-3.67)	11/11	1.81 (0.71-4.61)
General secondary education (80.2A)	8/7	1.79 (0.61-5.25)	8/7	2.48 (0.81-7.61)
**Health and social work (85)**	44/43	1.13 (0.69-1.86)	43/42	1.14 (0.68-1.91)
Activities for human health (85.1)	33/37	1.03 (0.60-1.79)	32/36	1.04 (0.59-1.84)
Hospital activities (85.1A)	28/32	1.00 (0.56-1.79)	27/31	0.97 (0.53-1.78)
Social action (85.3)	10/6	1.51 (0.51-4.46)	10/6	1.47 (0.47-4.57)

*Adjusted for sibship size, born from multiple pregnancy, personal history of testicular trauma, family history of testicular cancer and family history of cryptorchidism
