# Supplementary material for: Parental occupations at birth and risk of adult testicular germ cell tumors in offspring: a French nationwide case–control study
Source: Front Public Health. 2024 Jan 16;11:1303998. doi: 10.3389/fpubh.2023.1303998 (PMC10825020; doi:10.3389/fpubh.2023.1303998)
Supplement: Supplementary file 7 [file Data_Sheet_7.pdf]

Supplementary material

Table S7. Odds ratios (OR) and 95% confidence intervals (CI) for TGCT associated with father's job (ISCO-1968) and industry sector (NAF-1999) at birth, with additional adjustment for age at diagnosis(cases)/inclusion(controls), overall, case-control study, N=1124, France, 2015-2018.

	**N cases /<br>N controls**	**Adjusted OR*<br>(95% CI)**
**ISCO-1968 CODES**		
**Professional, Technical and Related Workers (0/1)**	83 / 173	0.75 (0.55-1.03)
Architects, engineers and related technicians (0-2/0-3)	29/57	0.90 (0.55-1.47)
Draughtsmen (0-32)	7/14	0.78 (0.30-2.03)
Electrical and electronics engineering (0-34)	5/7	0.81 (0.24-2.70)
Medical, dental, veterinary and related workers (0-6/0-7)	11/33	0.55 (0.27-1.13)
Medical doctors (0-61)	10/20	0.86 (0.39-1.91)
Statisticians and related technicians (0-8)	10/12	1.12 (0.46-2.72)
Systems analysts (0-83)	8/8	1.50 (0.54-4.17)
Systems analyst (0-83.10)	8/8	1.51 (0.54-4.25)
Accountants (1-1)	5/11	0.94 (0.32-2.78)
Accountants (1-10)	5/11	0.92 (0.31-2.71)
Teachers (1-3)	13/29	0.80 (0.40-1.63)
Secondary education teachers (1-32)	8/11	1.33 (0.50-3.57)
**Administrative and managerial workers (2)**	18/41	0.64 (0.35-1.15)

Managers (2-1)	15/41	0.50 (0.27-0.95)
Managers not elsewhere classified (2-19)	9/23	0.55 (0.24-1.23)
**Clerical and related workers (3)**	32/53	1.08 (0.67-1.73)
Bookkeepers, cashiers and related workers (3-3)	10/12	1.23 (0.50-3.01)
Bookkeepers and cashiers (3-31)	5/5	1.63 (0.46-5.86)
Bookkeepers, cashiers and related workers not elsewhere classified (3-39)	5/7	0.90 (0.26-3.10)
Finance clerk (3-39.40)	5/7	0.87 (0.25-3.00)
Mail distribution clerks (3-7)	5/5	2.10 (0.56-7.84)
Mail distribution clerks (3-70)	5/5	2.01 (0.54-7.49)
Clerical and related workers not elsewhere classified (3-9)	11/27	0.74 (0.36-1.52)
Correspondence and reporting clerks (3-93)	6/11	1.00 (0.36-2.81)
Office clerk (general) (3-93.10)	6/10	1.01 (0.35-2.90)
**Sales Workers (4)**	30/40	1.44 (0.86-2.39)
Working proprietors (wholesale and retail trade) (4-1)	7/8	2.16 (0.75-6.20)
Working proprietors (wholesale and retail trade) (4-10)	7/7	2.29 (0.78-6.77)
Working proprietor (retail trade) (4-10.30)	7/6	2.71 (0.88-8.36)
Technical salesmen, commercial travellers and manufacturers' agents (4-3)	11/18	1.14 (0.52-2.50)
Technical salesmen and service advisers (4-31)	10/15	1.17 (0.51-2.69)
Technical salesman (4-31.20)	10/15	1.16 (0.50-2.71)
Salesmen, shop assistants and related workers (4-5)	6/8	1.16 (0.38-3.49)

**Service Workers (5)**	34/33	1.63 (0.98-2.73)
Cooks, waiters, bartenders and related workers (5-3)	7/8	1.30 (0.45-3.76)
Protective service workers (5-8)	19/15	**2.05 (1.01-4.16)**
Protective service workers not elsewhere classified (5-89)	11/9	1.67 (0.67-4.15)
Other protective service workers (5-89.90)	8/9	1.14 (0.42-3.07)
**Agricultural, animal husbandry and forestry workers, fishermen and hunters (6)**	31/37	1.35 (0.80-1.38)
Farmers (6-1)	21/21	1.86 (0.97-3.58)
General farmers (6-11)	10/12	1.40 (0.56-3.49)
General farmer (6-11.10)	10/12	1.45 (0.58-3.65)
Specialized farmers (6-12)	11/8	**2.62 (1.01-6.82)**
Agricultural and animal husbandry workers (6-2)	6/10	0.93 (0.32-2.71)
**Production and Related Workers, Transport Equipment operators and labourers (7/8/9)**	146/233	1.05 (0.80-1.38)
Production supervisors and general foremen (7-0)	7/12	1.14 (0.43-2.99)
Production supervisors and general foremen (7-00)	7/12	1.10 (0.42-2.88)
Food and beverage processers (7-7)	8/16	0.73 (0.30-1.78)
Blacksmiths, toolmakers and machine-tool operators (8-3)	13/13	1.72 (0.76-3.88)
Machinery fitters, machine assemblers and precision-instrument makers [except electrical] (8-4)	15/33	0.67 (0.35-1.27)
Motor-vehicle mechanics (8-43)	5/10	0.69 (0.22-2.11)
Machinery fitters, machine assemblers and precision-instrument makers [except electrical] not elsewhere classified (8-49)	7/15	0.65 (0.26-1.65)
Electrical fitters and related electrical and electronics workers (8-5)	8/21	0.52 (0.22-1.22)

Plumbers, welders, sheet-metal and structural metal preparers and erectors (8-7)	11/21	0.95 (0.44-2.04)
Bricklayers, carpenters and other construction workers (9-5)	20/37	0.94 (0.53-1.68)
Bricklayers, stonemasons and tile setters (9-51)	13/13	1.72 (0.77-3.85)
Material handling and related equipment operators, dockers and freight handlers (9-7)	5/19	0.36 (0.13-1.03)
Transport equipment operators (9-8)	35/29	**2.06 (1.22-3.50)**
Motor-vehicle drivers (9-85)	31/27	**1.78 (1.03-3.09)**
Lorry and van driver (local transport). (9-85.50)	13/11	1.66 (0.71-3.88)
Lorry and van driver (long-distance transport) (9-85.60)	5/8	0.88 (0.27-2.88)
**NAF-1999 CODES**		
**Agriculture, hunting and forestry (01, 02)**	31/38	1.36 (0.81-2.29)
Agriculture, hunting and related service activities (01)	27/32	1.55 (0.89-2.71)
Growing of crops combined with farming of animals (mixed farming) (01.3)	10/12	1.29 (0.52-3.21)
Growing of crops combined with farming of animals (mixed farming) (01.3Z)	10/12	1.30 (0.52-3.25)
**Manufacturing (15 to 37)**	56/103	0.81 (0.56-1.17)
Food industry (15)	6/13	0.61 (0.22-1.69)
Publishing, printing, reproduction (22)	8/5	3.10 (0.97-9.89)
Metalworking (28)	15/23	1.04 (0.52-2.07)
**Electricity, gas and water supply (40, 41)**	5/5	1.77 (0.48-6.47)
**Construction (45)**	41/69	1.04 (0.68-1.59)
Construction of building or civil engineering works (45.2)	13/23	0.99 (0.48-2.04)

Installation works (45.3)	6/17	0.61 (0.23-1.59)
Building completion work (45.4)	14/13	1.81 (0.82-4.00)
**Wholesale and retail trade ; repair of motor vehicles,motorcycles and personal and household goods (50, 51, 52)**	42/62	1.13 (0.73-1.74)
Sale and repair of motor vehicles (50)	10/13	1.13 (0.47-2.69)
Maintenance and repair services of motor vehicles (50.2)	8/11	1.07 (0.41-2.80)
Maintenance and repair services of motor vehicles (50.2Z)	8/11	1.05 (0.40-2.79)
Wholesale trade and trade intermediaries (51)	7/13	0.92 (0.36-2.37)
Retail and repair of household goods (52)	25/36	1.20 (0.69-2.07)
Food retailing in specialized stores (52.2)	7/10	1.20 (0.44-3.29)
Other retail in specialized stores (52.4)	11/12	1.50 (0.64-3.56)
**Hotels and restaurants (55)**	11/13	1.28 (0.55-2.97)
Restaurants (55.3)	5/9	0.84 (0.27-2.59)
**Transport, storage and communication (60, 61, 62, 63, 64)**	42/59	1.17 (0.75-1.80)
Land transport (60)	29/34	1.40 (0.82-2.37)
Transport via railways (60.1)	6/9	1.24 (0.43-3.60)
Transport via railways (60.1Z)	6/9	1.24 (0.43-3.59)
Urban and road transport (60.2)	21/24	1.77 (0.73-2.56)
Local road transport of goods (60.2L)	8/8	1.30 (0.47-3.66)
Post and telecommunications (64)	8/19	0.64 (0.26-1.54)
Post and courier activities (64.1)	5/8	1.09 (0.33-3.60)

National post activities (64.1A)	5/8	1.09 (0.33-3.58)
**Financial intermediation (65, 66, 67)**	10/18	0.79 (0.35-1.81)
Financial intermediation (65)	9/15	0.87 (0.36-2.09)
Monetary intermediation (65.1)	9/15	0.87 (0.36-2.09)
**Real estate, renting and business activities (70, 71, 72, 73, 74)**	19/42	0.71 (0.39-1.28)
Services provided primarily to businesses (74)	13/23	0.86 (0.41-1.77)
Architectural and engineering activities (74.2)	6/19	0.48 (0.18-1.26)
Engineering, technical studies (74.2C)	5/14	0.58 (0.20-1.67)
**Public administration and defence; compulsory social security (75)**	36/45	1.47 (0.91-2.37)
General, economic and social administration (75.1)	15/14	2.09 (0.97-4.52)
General public administration (75.1A)	10/13	1.52 (0.64-3.62)
Public prerogative services (75.2)	21/31	1.11 (0.61-2.02)
Defense (75.2C)	13/24	0.78 (0.38-1.61)
Police (75.2G)	16/31	0.92 (0.48-1.78)
**Education (80)**	16/31	0.92 (0.48-1.78)
Secondary education (80.2)	10/11	1.63 (0.64-4.16)
General secondary education (80.2A)	19/41	0.70 (0.39-1.25)
**Health and social work (85)**	19/41	0.70 (0.39-1.25)
Activities for human health (85.1)	16/32	0.77 (0.40-1.46)
Hospital activities (85.1A)	7/12	1.02 (0.38-2.72)

Medical practice (85.1C)	6/13	0.70 (0.25-1.95)
**Other community, social and personal services activities (90, 91, 92, 93)**	6/13	0.89 (0.33-2.40)

*Adjusted for sibship size, born from multiple pregnancy, personal history of testicular trauma, family history of testicular cancer, family history of cryptorchidism and age at diagnosis (cases)/ inclusion (controls).
