# Supplementary material for: Parental occupations at birth and risk of adult testicular germ cell tumors in offspring: a French nationwide case–control study
Source: Front Public Health. 2024 Jan 16;11:1303998. doi: 10.3389/fpubh.2023.1303998 (PMC10825020; doi:10.3389/fpubh.2023.1303998)
Supplement: Supplementary file 8 [file Data_Sheet_8.pdf]

Supplementary material

Table S8. Odds ratios (OR) and 95% confidence intervals (CI) for TGCT associated with mother's job (ISCO-1968) and industry sector (NAF-1999) at birth, with additional adjustment for age at diagnosis(cases)/inclusion(controls), overall, case-control study, N=1124, France, 2015-2018.

	**N cases / N controls**	**Adjusted OR* (95% CI)**
**ISCO-1968 CODES**		
**Professional, Technical and Related Workers (0/1)**	85/137	1.03 (0.75-1.41)
Medical, dental, veterinary and related workers (0-6/0-7)	23/50	0.72 (0.43-1.23)
Medical doctors (0-61)	5/7	1.28 (0.39-4.28)
Professional Nurses (0-71)	13/24	0.89 (0.44-1.81)
Accountants (1-1)	15/13	1.70 (0.79-3.68)
Accountants (1-10)	15/13	1.70 (0.79-3.67)
Auditor (1-10.20)	15/11	2.05 (0.91-4.60)
Teachers (1-3)	31/41	1.37 (0.83-2.28)
Secondary education teachers (1-32)	18/14	**2.94 (1.40-6.18)**
Primary Education Teachers (1-33)	10/19	0.78 (0.35-1.77)
First-Level Education Teacher (1-33.20)	10/19	0.80 (0.36-1.82)
Professional, technical and related workers not elsewhere classified (1-9)	7/17	0.66 (0.26-1.65)
Social workers (1-93)	5/7	0.98 (0.30-3.21)
**Administrative and Managerial Workers (2)**	5/11	0.58 (0.19-1.75)

Managers (2-1)	5/11	0.58 (0.19-1.75)
Managers not elsewhere classified (2-19)	5/9	0.69 (0.22-2.14)
**Clerical and Related Workers (3)**	86/124	1.16 (0.83-1.60)
Stenographers, typists and card-and tape-punching machine operators (3-2)	27/45	0.98 (0.59-1.65)
Stenographers, Typists and Teletypists (3-21)	27/45	0.98 (0.58-1.65)
Stenographer-Typist (General) (3-21.10)	19/32	0.94 (0.50-1.75)
Stenographic Secretary (3-21.20)	9/13	1.15 (0.48-2.81)
Bookkeepers, cashiers and related workers (3-3)	6/19	0.59 (0.23-1.52)
Bookkeepers and cashiers (3-31)	5/13	0.74 (0.25-2.14)
Clerical and related workers not elsewhere classified (3-9)	40/49	1.28 (0.81-2.02)
Correspondence and reporting clerks (3-93)	34/41	1.33 (0.81-2.19)
Office clerk (general) (3-93.10)	29/35	1.35 (0.79-2.31)
**Sales Workers (4)**	21/35	1.00 (0.56-1.79)
Salesmen, shop assistants and related workers (4-5)	12/19	1.02 (0.47-2.19)
Salesmen, shop assistants and demonstrators (4-51)	12/18	1.08 (0.50-2.34)
Retail trade salesman (4-51.30)	7/15	0.82 (0.32-1.68)
**Service Workers (5)**	42/73	1.88 (0.58-1.33)
Cooks, waiters, bartenders and related workers (5-3)	6/12	0.78 (0.28-2.16)
Maids and related housekeeping service workers not elsewhere classified (5-4)	8/6	2.02 (0.67-6.06)
Maids and related housekeeping service workers not elsewhere classified (5-40)	8/6	2.01 (0.67-6.02)

Building caretakers, charworkers, cleaners and related workers (5-5)	10/21	0.69 (0.31-1.50)
Charworkers, cleaners and related workers (5-52)	10/20	0.74 (0.33-1.62)
Charworker (5-52.20)	10/20	0.76 (0.35-1.68)
Service workers not elsewhere classified (5-9)	11/20	0.87 (0.40-1.87)
Other service workers (5-99)	11/20	0.87 (0.40-1.88)
Nursing Aid (5-99.40)	11/19	0.92 (0.42-2.02)
**Agricultural, Animal Husbandry and Forestry Workers, fishermen and hunters (6)**	9/14	1.22 (0.50-2.97)
Farmers (6-1)	8/10	1.47 (0.55-3.97)
Specialized farmers (6-12)	5/7	1.28 (0.37-4.43)
**Production and Related Workers, Transport Equipment operators and labourers (7/8/9)**	16/44	0.56 (0.31-1.02)
Tailors, dressmakers, sewers, upholsterers and related workers (7-9)	5/12	0.73 (0.25-2.15)
**NAF-1999 CODES**		
**Agriculture, hunting and forestry (01, 02)**	9/14	1.17 (0.48-2.86)
Agriculture, hunting and related service activities (01)	9/14	1.17 (0.48-2.86)
**Manufacturing (15 to 37)**	18/43	0.66 (0.37-1.19)
Clothing and fur industry (18)	6/10	1.00 (0.35-2.85)
Manufacture of textile clothing (18.2)	6/10	1.02 (0.36-2.91)
**Wholsale and retail rade; repair of motor vehicls, motorcycles and personal and household goods (50, 51, 52)**	24/43	0.91 (0.53-1.56)
Retail and repair of household goods (52)	21/39	0.91 (0.51-1.60)
Other retail in specialized stores (52.4)	8/11	1.10 (0.42-2.86)

**Hotels and restaurants (55)**	10/19	0.76 (0.34-1.69)
Restaurants (55.3)	7/12	0.91 (0.35-2.38)
**Transport, storage and communication (60, 61, 62, 63, 64)**	8/13	0.81 (0.32-2.05)
**Financial intermediation (65, 66, 67)**	6/15	0.71 (0.27-1.91)
**Real estate, renting and business activities (70, 71, 72, 73, 74)**	15/19	1.15 (0.55-2.40)
Services provided primarily to businesses (74)	9/11	1.00 (0.39-2.57)
Legal, accounting and management consulting activities (74.1)	7/7	1.21 (0.40-3.67)
**Public administration and defence; compulsory social security (75)**	16/33	0.85 (0.45-1.61)
General, economic and social administration (75.1)	10/22	0.74 (0.34-1.64)
General public administration (75.1A)	8/20	0.64 (0.27-1.53)
**Education (80)**	35/47	1.33 (0.82-2.14)
Primary education (80.1)	14/22	0.93 (0.45-1.90)
Primary education (80.1Z)	14/22	0.89 (0.43-1.83)
Secondary education (80.2)	18/14	**2.84 (1.35-5.97)**
General secondary education (80.2A)	8/6	2.71 (0.89-8.24)
**Health and social work (85)**	49/91	0.81 (0.55-1.20)
Activities for human health (85.1)	28/62	0.75 (0.46-1.22)
Hospital activities (85.1A)	21/36	0.95 (0.53-1.69)
Social action (85.3)	10/16	0.88 (0.39-2.00)
**Other community, social and personal services activities (90, 91, 92, 93)**	8/14	0.96 (0.38-2.41)

[*]Adjusted for sibship size, born from multiple pregnancy, personal history of testicular trauma, family history of testicular cancer, family history of cryptorchidism and age at diagnosis (cases)/ inclusion (controls).
