# Supplementary figures and images for: Parental occupations at birth and risk of adult testicular germ cell tumors in offspring: a French nationwide case–control study
Source: Front Public Health. 2024 Jan 16;11:1303998. doi: 10.3389/fpubh.2023.1303998 (PMC10825020; doi:10.3389/fpubh.2023.1303998)

Figure S1. Flow chart of the TESTIS population

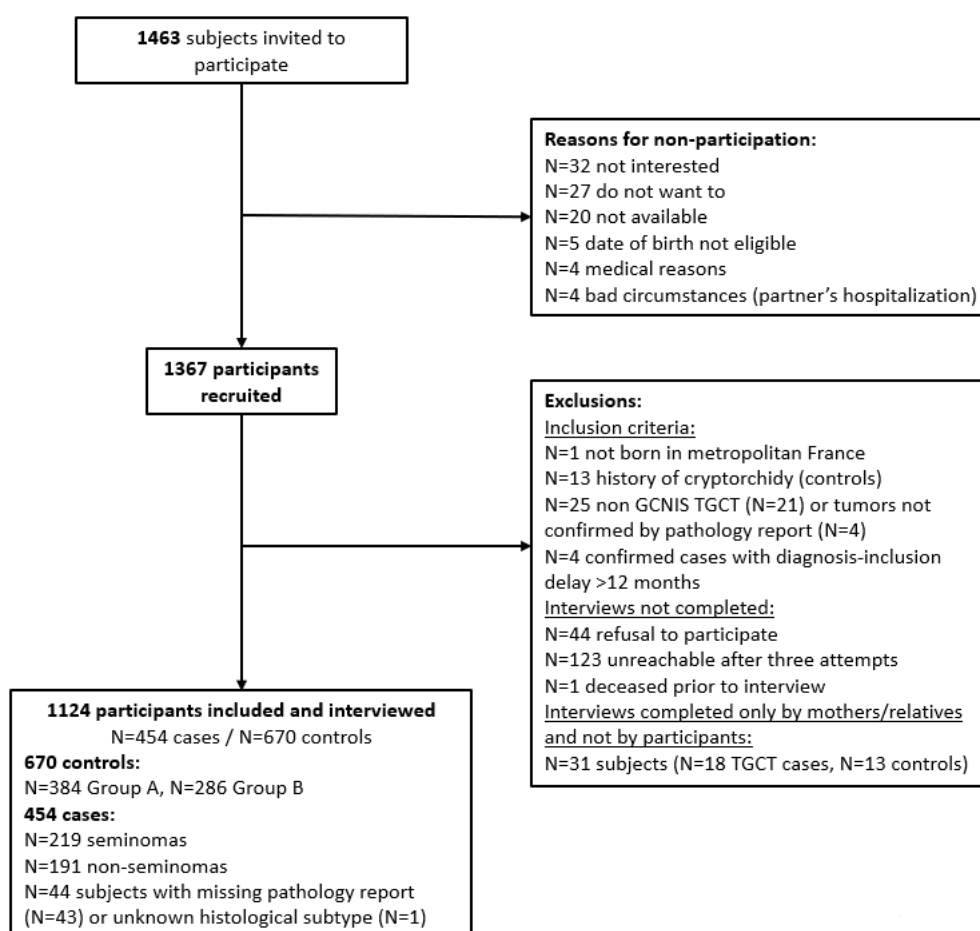

Supplement: Supplementary file 9 [file Presentation_1.pdf]
